# The risk of re-identification remains high even in country-scale location datasets

**DOI:** 10.1016/j.patter.2021.100204

**Published:** 2021-03-12

**Authors:** Ali Farzanehfar, Florimond Houssiau, Yves-Alexandre de Montjoye

**Affiliations:** 1 Department of Computing, Imperial College London, London SW7 2AZ, UK

**Keywords:** privacy, anonymisation, de-identification, unicity, re-identification, call detail records, data protection, human mobility, location data, applied mathematics

## Abstract

Although anonymous data are not considered personal data, recent research has shown how individuals can often be re-identified. Scholars have argued that previous findings apply only to small-scale datasets and that privacy is preserved in large-scale datasets. Using 3 months of location data, we (1) show the risk of re-identification to decrease slowly with dataset size, (2) approximate this decrease with a simple model taking into account three population-wide marginal distributions, and (3) prove that unicity is convex and obtain a linear lower bound. Our estimates show that 93% of people would be uniquely identified in a dataset of 60M people using four points of auxiliary information, with a lower bound at 22%. This lower bound increases to 87% when five points are available. Taken together, our results show how the privacy of individuals is very unlikely to be preserved even in country-scale location datasets.

## Highlights

• Re-identification risk is statistically modeled and shown to decrease slowly with dataset size
• With increasing dataset size, the unicity decrease is lower-bounded and convex
• Previous estimates of unicity unrealistically underestimated the risks
• Individuals are likely re-identifiable in country-size location data and other high-dimensional datasets

## The bigger picture

Data about us are being collected in many different ways, when we use our bank cards, use our phones, browse the web, or even drive our cars. These datasets contain detailed information about our lives. For each person, a dataset might contain thousands to tens of thousands of records. Previous research has shown that knowing just a few points about a target can single out the vast majority of people in location datasets. However, some had argued the risk of re-identification becomes negligible if we look at large-scale datasets containing tens of millions of people.

Here, we empirically measure, mathematically model, and provide a lower bound on the relationship between the size of a dataset and the risk of re-identification. Our results all show that re-identification risk decreases very slowly with increasing dataset size. Contrary to previous claims, people are thus very likely to be re-identifiable even in country-scale datasets.

## Teaser

Researchers have claimed that individuals could not be re-identified in large-scale location datasets, making them safe. We here empirically measure and mathematically model the relationship between the size of a dataset and the risk of re-identification. Our results show that the risk decreases slowly with dataset size, making even large country-scale datasets very likely to be re-identifiable.

## Introduction

Throughout our day, we interact with many digital services when using our phone, paying with our credit card, or using public transport with a smart card. This results in our location data being collected broadly, sometimes on the scale of countries. For instance, Vodafone UK collects location trajectories of 20M citizens^1^—a third of the population—while up to 5 million people use London's subway daily.^2^

Location data have been used extensively in research. In urban planning, mobility data can be used to monitor urban activity^3^ and help design better cities.^4^ In epidemiology, it has been used to monitor and mitigate the spread of infectious diseases such as Ebola and COVID-19.^5^, ^6^, ^7^, ^8^, ^9^, ^10^ In computational social science, it has allowed us to gain unprecedented insights into the spatial distribution of poverty,^11^ and even to study the impact of mass employment layoffs on society.^12^ Further, the use of location data has withstood scrutiny into potential biases in their collection mechanisms.^13^

Despite this, the large-scale collection and use of location data has raised serious privacy concerns. It consists of fine-grained records of where we are and how we move around, and was considered sensitive by 82% of Americans in a recent survey.^14^ Location data can furthermore be used to predict individuals' income,^11^^,^^15^ their home and work locations,^16^, ^17^, ^18^, ^19^, ^20^, ^21^ when they sleep and wake up,^22^, ^23^, ^24^, ^25^, ^26^ their gender and age,^27^ their personality,^28^ who their friends are,^29^^,^^30^ and where they tend to socialize.^31^

Unicity has been proposed as a measure for the risk of re-identification in anonymous datasets and was used to show how four points of auxiliary information (places and times where someone was) are enough to uniquely identify 95% of people in a large-scale location dataset.^32^ These four points of auxiliary information could be in the form of geo-tagged “tweets,” online check-ins, or information obtained by more traditional means, such as observing someone making a call. Unicity ($\epsilon_{p}$) is defined as the fraction of trajectories that are unique based on knowledge of *p* randomly chosen points in a given trajectory. Unicity has since been used to quantify re-identification risk across a number of domains, including the mobility of vehicles,^33^ apps downloaded by smartphones over time,^34^^,^^35^ smart cards used in public transport,^24^ credit card transaction histories,^36^ and location data from mobile phones in a number of countries.^32^^,^^37^^,^^38^ A range of studies have furthermore exploited the unicity of datasets to re-identify people. Narayanan and Shmatikov famously showed that close to 90% of people could be re-identified in the Netflix dataset,^39^ while Riederer and colleagues used the unicity of traces to match the same individual across multiple datasets.^40^

Researchers and industry practitioners have, however, argued that these high unicity numbers are an artifact of the small size of the datasets considered, and are overestimating the risk of re-identification.^41^, ^42^, ^43^ For instance, Riederer et al.^40^ relied on a location dataset of 1.7k people, while other case studies report unicity on dataset sizes ranging from several thousands (respectively 12k and 55k)^33^^,^^34^ to over 1 million people (1.5M).^32^ Examining a published study,^36^ El Emam et al. estimated that the unicity of a dataset of ≈20M trajectories will be as low as 1% given four points of auxiliary information, the conclusion being that privacy was preserved in such large datasets.^42^

We here (1) study 3 months of location data and show empirically that unicity decreases slowly with the size of the dataset, (2) approximate this decrease with a simple statistical model taking into account three population-wide marginal distributions along with the underlying geography, and (3) prove that the decrease in unicity is a convex function of the dataset size and obtain a linear lower bound on unicity. We finally perform a sensitivity analysis suggesting that the decrease in unicity is agnostic to broad perturbations in the input distributions. These results disprove previous claims, instead showing that unicity is likely to remain high even in country-scale datasets.

## Results

Our experiments are performed on a dataset of call detail records containing the location of 1M individuals over 3 months. Each record contains a unique user ID, an hourly time stamp, and an antenna ID, which relates to a location (see Supplemental information for more details). We formally model this dataset as a sequence, $D=\left(D_{1},\ldots ,D_{N}\right)$, populated with user time/location traces of the type $D_{i}=\left(X_{i},C_{i}\right)$. $X_{i}$ and $C_{i}$ are lists of positions (antennas) and times (hours) representing the spatial and temporal components of a user's location trace.

Using this dataset, we empirically study the decrease in unicity with the dataset size by randomly sampling individuals from our original dataset and measuring the unicity of the sample as we increase its size (see Experimental procedures for details). We use the formal definition of unicity and the estimation algorithm S2 from de Montjoye et al.^36^ In line with previous work, we use the subscript *p* in $\epsilon_{p}\left(N\right)$ to indicate the number of points of auxiliary information used in the computation of unicity.

**Figure 1:**
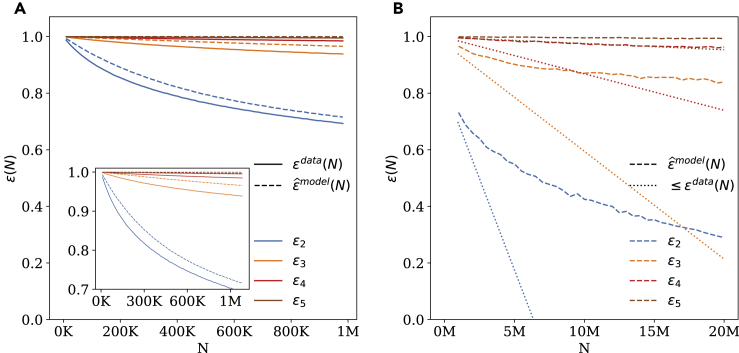
The relationship between unicity and dataset size (A) Empirical (solid lines) and estimated (dashed lines) unicity decreases slowly with the size of the dataset. Inset: close up of the region ϵ≥0.7. (B) The estimated unicity remains high even in large datasets. This is confirmed by the lower bound results (dotted lines). Taken together, these results strongly suggest that unicity remains high even in country-scale datasets.

Figure 1A shows that unicity empirically decreases slowly with the size of the dataset. With three points of auxiliary information, unicity (solid orange line) goes down from $\epsilon_{3}\left(100\text{K}\right)=0.98$ in a dataset of 100,000 people to $\epsilon_{3}\left(1\text{M}\right)=0.93$ in a dataset of a million people. With two points (solid blue line) this decreases slightly faster, reaching $\epsilon_{2}\left(1\text{M}\right)=0.69$, while unicity with four points or more (solid red and brown lines) decreases very slowly with $\epsilon_{4}\left(1\text{M}\right)=0.98$. These results show that, while the size of the dataset has an impact on unicity, the decrease in unicity is slow.

To further study how unicity decreases with dataset size and whether it decreases sufficiently in population-scale datasets, we propose a simple statistical model taking into account three population-wide marginal distributions—circadian $\left(P_{C}\right)$, frequency $\left(P_{F}\right)$, and activity $\left(P_{A}\right)$—along with the network of mobile phone antennas in a country. Using solely these quantities, the model is able to replicate the observed decrease in unicity with dataset size.

**Figure 2:**
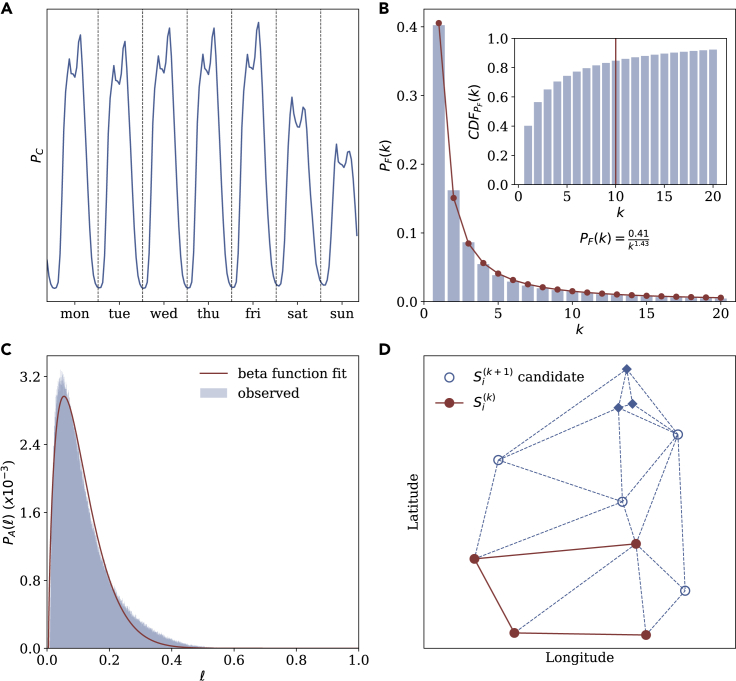
Inputs to the unicity model (A) The circadian distribution, $P_{C}$. (B) The frequency distribution, $P_{F}$, along with a power law fit (solid line, $R^{2}=0.99$). The inset displays the cumulative distribution with 85% of activity captured by the top 10 locations. (C) The activity distribution, $P_{A}$, indicating the distribution of the number of records per trajectory along with a β distribution fit (solid line, $R^{2}=0.98$). (D) Illustration of the sub-graph sampling method used to generate an antenna set $S_{i}$ where $S_{i}^{\left(k\right)}\in S_{i}$. The underlying antenna network is represented by dotted lines. The filled nodes (circles) correspond to locations already selected, while the hollow nodes are potential locations that could be selected next ($S_{i}^{\left(k+1\right)}$ candidates) (see Supplemental information for detailed algorithm). Remaining locations are represented by filled diamonds.

Figure 2 displays the information extracted from the dataset, three distributions, and the antenna network. $\left(P_{C}\right)$ characterizes the circadian cycle, the overall likelihood of a record to occur at a given time in a week. The existence of circadian cycles is well documented in the computational social science literature,^22^^,^^23^^,^^25^^,^^26^ and we use their empirical form in the model. The frequency distribution, $\left(P_{F}\right)$, is the relative overall likelihood of a location to be visited. This distribution too has been studied before and has been widely shown to be well approximated by a power-law distribution,^44^, ^45^, ^46^, ^47^, ^48^ as is also the case here (Figure 2B, $R^{2}=0.99$). The activity distribution, $\left(P_{A}\right)$, captures the number of records $\ell_{\left(i\right)}=\left|D_{i}\right|$ that appear in each user trace. We approximate it here with a β distribution (α=1.72, β = 14.7, $R^{2}=0.98$). Finally, $S_{i}$ is the set of locations visited by person *i*. It is a sub-graph sampled from the Delaunay tessellation of the antenna coordinates (L) in the dataset (see Supplemental information for the detailed algorithm).

In short, for each user, our model samples a list of 10 connected antennas $\left(S_{1},\ldots ,S_{10}\right)$ on the network and an activity (number of records in the user's trace), $A∼P_{A}$. Each record's timestamp *C* and position *X* is then sampled according to the circadian distribution $C∼P_{C}$ and $X=S_{K},\;K∼P_{F}$. This model is formally defined in the Experimental procedures.

Figure 1A shows that our simple statistical model closely follows the empirical measure of unicity from 1 to 1M people (dashed and solid lines). Using the model, we then study how unicity is likely to evolve as the size of the dataset increases to 20M people (Figure 1B). For N=20M, our model estimates unicity with three points to be close to $\hat{\epsilon_{3}}\left(20\text{M}\right)=0.93$, while knowing one more point would increase this to the region of $\hat{\epsilon_{4}}\left(20\text{M}\right)=0.99$. This is a stark difference with the linear extrapolation made by El Emam,^42^ who reports a unicity of 0.01 with four points (we replicate El Emam's method in the discussion and display our results for up to 60M people in the Supplemental information).

The model provides good evidence that unicity is likely to remain high even in datasets as large as 20M people. For further evidence, we prove that the decrease in unicity with increasing dataset size follows a convex form, and use this result to provide a lower bound on unicity in large datasets. We show in the Supplemental information that the unicity of a dataset of size *N* can be expressed as a sum of convex functions of *N*, and is thus convex.

This builds on two assumptions: (1) there exists an underlying trajectory distribution $T_{X}$ from which all trajectories $D_{i}\in D$ are sampled and (2) all trajectories are independent of one another, $D_{i}⊥⊥D_{j}$. The first assumption states that an underlying distribution for trajectories exists. Such a distribution would also capture correlations between individuals on a large scale (e.g., commuting patterns, cities, weekends). The second assumption presumes that the correlation between specific individuals is negligible when estimating unicity of large datasets.

A direct consequence of unicity being a strictly decreasing convex function is that it will be lower bounded by its linear tangent (treating unicity as a function of a real-valued *N*):

(Equation 1) $$\epsilon \left(D\left(N\right)\right)\geq \epsilon \left(D\left(N^{\prime }\right)\right)+\left(N-N^{\prime }\right)\cdot {\frac{d\epsilon }{dN}|}_{N=N^{\prime }}\text{.}$$

Re-arranged and expressed for discrete values, this gives a lower bound for unicity:

(Equation 2) $$\epsilon \left(D\left(N^{\prime }\right)\right)-\epsilon \left(D\left(N\right)\right)\leq \left(N-N^{\prime }\right)\cdot \left(\epsilon \left(D\left(N^{\prime }-1\right)\right)-\epsilon \left(D\left(N^{\prime }\right)\right)\right)\text{.}$$

Using the tangent to the empirical unicity curves estimated by discrete difference over the range of N∈[0.9M,1M], we obtain a lower bound of 0.73 for $\epsilon_{4}\left(20\text{M}\right)$ and 0.9 for $\epsilon_{5}\left(20\text{M}\right)$ (Figure 1B, dotted lines).

Our results show that unicity decreases slowly with the size of the dataset and that it, very likely, remains high even in population-scale datasets. This refutes previous claims that privacy is preserved in population-scale datasets, instead showing the risk of re-identification to be high. Modern location datasets have a great potential to improve our society, for example, by training AI algorithms, but robust privacy engineering solutions are needed to use them safely.

## Discussion

Taken together, these results show that the scale of a dataset does not prevent re-identification. Human mobility, much like a physical fingerprint, is highly unique and can be used to find a person across mobility datasets.

Legally, the European Union (EU) General Data Protection Regulation sets a high threshold for what constitutes anonymous data, namely that the individual should not be identifiable taking into account both the “available technology at the time of the processing” but also future “technological developments” (Recital 26). The Article 29 Working Party, the predecessor to the European Data Protection Board, in its guidance sets out three criteria to assess whether a dataset is anonymous, singling out, linkability, and inference^49^ with the former two being directly applicable here. As an example, the Centre for Humanitarian Data of the United Nations (UN OCHA) adopted 5% as a threshold for what constitutes an acceptable re-identification risk.^50^ Even our lower bound of 22% far exceeds this liberal threshold.

Finally, here we study the unicity of location datasets with a spatial resolution of ≈1 km^2^ and a temporal resolution of an hour. Fine-grained GPS data are likely to lead to even higher values of unicity, and previous research has shown that, in general, de-identification methods do not meaningfully reduce the risk of re-identification. For instance, research^32^^,^^34^ has shown that reducing the spatial and temporal resolution of the data further only slowly decreases the risk, while another study^51^ concluded that location data “show poor anonymizability [as measured by k-anonymity], i.e., require important spatial and temporal generalization in order to slightly improve user privacy".

Ensuring that these data can be accessed and used broadly is of paramount importance, but this should not come at the expense of people's privacy. A range of privacy engineering techniques allowing data to be used while giving individuals strong privacy guarantees have been developed and are starting to be used.^52^, ^53^, ^54^ As standards for anonymization are being redefined, in the EU and around the world, it is essential for them to emphasize the strong limits of de-identification, possibly banning the uncontrolled release of individual-level de-identified data, and to give guidance on the use of modern privacy-engineering solutions.

In the next three sections we discuss the underlying assumptions of the unicity model and some considerations regarding the sensitivity of our results and, finally, include a discussion on previous estimates of unicity.

### Assumptions underpinning the simple unicity model

We here evaluate the four assumptions underpinning the simple unicity model we present.

First, the model treats each of the four inputs in Figure 2 as independent of one another. Considering them, or some of them, jointly might further improve the model. This would, however, also increase its complexity and, therefore, its sensitivity to small changes in the data. Although further exploration would be interesting, we consider that the simple model approximates the decrease in unicity with increasing dataset size well enough to support our conclusion that unicity is unlikely to be low even in population-scale datasets.

Second, our model uses input distributions extracted from a dataset of 1M people to study the unicity of datasets with up to 60M people (see Supplemental information). This assumes that these distributions estimated from a smaller sample are representative of the larger sample (i.e., the estimation of the distributions has converged). We show that this is a reasonable assumption by instantiating our model M with distributions extracted from samples of sizes significantly smaller than 1M, and showing that the unicity results remain largely unchanged (Figure S5 in Supplemental information). We also perform a sensitivity analysis to evaluate the impact of broad variations on these input distribution on our results (see next subsection).

Third, the model assumes each trajectory to contain at most one unique location. This allows for the mean frequency distribution ($P_{F}$) to be used in the modeling process (Figure 2B). As seen in the inset of Figure 2B, more than 85% of the activity in the average trajectory is captured by the top 10 locations visited. Furthermore, we find that $P_{F}$ changes only slightly when the number of unique locations is altered, and that our conclusions are not influenced by this choice.

Finally, our model assumes that the set of locations appearing in each trajectory can be described by a connected planar sub-graph of the underlying antenna network. We believe this to be a reasonable assumption, as previous work suggests that sub-graphs spanned by each trajectory in human mobility are highly localized, with the distribution $P\left(r_{g}\right)$ of the radius of gyration—a metric for how far people tend to travel on average—following a power law with increasing radius.^55^

### Sensitivity analysis

Our simple statistical model for unicity takes as input three distributions. However, these distributions may vary depending on specifics of the dataset, such as the country where it was collected or the sources of location information. Here we perform a sensitivity analysis to ensure the robustness of our model to even broad changes to the distributions.

**Figure 3:**
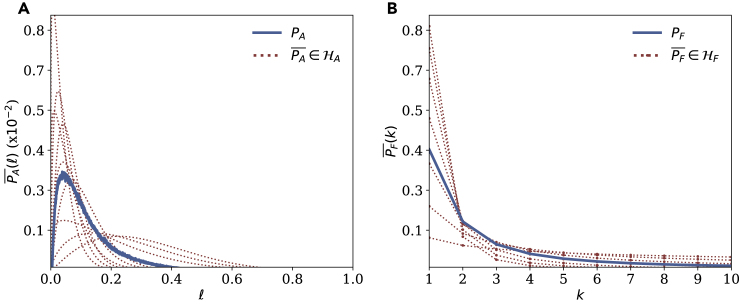
Range of distributions studied for the sensitivity analysis The ranges of perturbed activity $\bar{P_{A}}$ (A) and frequency $\bar{P_{F}}$ (B) distributions are displayed (dotted lines) along with their empirical forms (solid lines).

We first perturb the $P_{A}$ and $P_{F}$ distributions (Figure 3) around their empirical forms using a scaled earth mover’s distance as the guiding metric (see Supplemental information for details). The $P_{C}$ distribution, on the other hand, has been shown to be very stable across datasets^22^, ^23^, ^24^, ^25^, ^26^ and we thus keep it constant throughout our analysis.

These distributions are combined to produce 63 different instantiations of the unicity model (Figure S2). Table 1 summarizes the unicity values for models using the broad range of distributions in Figure 3, at a dataset size of 20M trajectories (see Supplemental information for 60M results). Note that the lowest unicity values across all instantiations of the model are still high, with $Min\left(\epsilon_{4}\left(20M\right)\right)\;=\;43.1\text{\%}$ and would still be considered as putting people's privacy at risk.

**Table 1:** Summary of unicity results at N=20M as per the sensitivity analysis

	$\epsilon_{2}$	$\epsilon_{3}$	$\epsilon_{4}$	$\epsilon_{5}$
Mean	0.307	0.735	0.876	0.935
Standard deviation	0.175	0.216	0.159	0.113
Minimum	0.071	0.260	0.431	0.544
Maximum	0.704	0.997	1	1

Further, we study how certain aspects of human mobility contribute to unicity. Starting from empirical user location traces $D_{i}=\left(X_{i},C_{i}\right)$, first, we find that removing the association between times ($C_{i}$) and locations ($X_{i}$), by shuffling the vectors and recombining them, only slightly affects unicity values (Figure S4A). Specifically, consider a dataset $D^{\prime }$ composed of trajectories $D{\text{′}}_{i}=\left(X{\text{′}}_{i},C{\text{′}}_{i}\right)$ such that:

$$X{\text{′}}_{i}=\sigma_{i}\left(X_{i}\right)\text{,}$$

$$C{\text{′}}_{i}=\pi_{i}\left(C_{i}\right)\text{,}$$

where $\sigma_{i}$ and $\pi_{i}$ refer to random permutations of the spatial and temporal components of $D_{i}$. This only marginally affects unicity, showing that unicity does not depend on the specific places being visited at specific times, as long as those times and places appear in the trace with their respective frequencies independently.

Second, we replace the set of locations in each trajectory with uniformly picked locations. Instead of using the sub-graph sampling method displayed in Figure 2D, we populate each $S_{i}$ with antennas picked from the entire set of locations L uniformly at random. We find that this leads to unicity being overestimated (Figure S4D).

Third, replacing $P_{C}$ or $P_{F}$ with uniform distributions (Figures S4B and S4C) or attempts to model unicity using a simple combinatorial model (Figure S3) also cause the model to overestimate unicity. These demonstrate the importance of all three distributions and the underlying geography to correctly capture the unicity of mobility datasets.

This analysis, combined with the relative simplicity and generality of the unicity model, strongly suggest that our results would generalize to any location dataset. Likewise, the strong underlying combinatorial effect that underpins unicity combined with previous research^34^, ^35^, ^36^ suggests that unicity will similarly decrease slowly in other types of high-dimensional data.

### El Emam's method

El Emam^42^ proposed a method (hereafter the EE method) to estimate the uniqueness of a population-size (*N*) dataset given the unicity ϵ(m) of a smaller sample dataset of size *m*. Using this method, he estimates that the uniqueness for a population of size $N=22\cdot 10^{6}$ is about 1%, given a uniqueness of 90% of a sample of size $m=22\cdot 10^{6}$ of the same dataset. This estimate forms the basis for his claim that uniqueness is low in large-scale datasets.

We here show that the EE method (1) is unrealistic and (2) provably gives the lowest possible estimate for the risk in the larger dataset, and that (3) by using our dataset, we observe that the real empirical unicity is significantly higher than the upper bound given by the EE method.

First, the method is unrealistic, as it effectively generates a dataset *D* of size *N* where a fraction α of records are unique, while all the other records are identical to exactly one and only one other record. The parameter α is selected such that the expected estimated uniqueness on a sample of size *m*, which we denote by $\nu_{D}\left(m\right)$, is equal to the empirical unicity. This assumes that users in the real mobility dataset are either unique or exact duplicates of another user.

Second, we prove in the Supplemental information that the risk estimated by the EE method will be lower or equal to the risk of *any* other dataset of size *N*, as this estimate is an *affine* function of *m*. In other terms, this method will *always* return the absolute lowest possible estimate of the risk.

Third, we apply the EE method to our dataset and show that its estimate of the risk is significantly lower than the real empirical value, leading to the risk of re-identification being strongly underestimated. For a dataset of 200,000 people, we empirically observe an $\epsilon_{2}\left(200K\right)=0.86$. Using this number, El Emam's method would estimate the risk of a larger 1M person dataset to be $\epsilon_{2}\left(1M\right)=0.3$, while the correct empirical value is ≈0.7.

Taken together, our results cast serious doubt on the validity of the EE method to carry out risk assessments.

## Experimental procedures

### Resource availability

#### Lead contact

Further information and requests for resources should be directed to and will be fulfilled by the lead contact, Yves-Alexandre de Montjoye (demontjoye@imperial.ac.uk).

#### Materials availability

There are no physical materials associated with this study.

#### Data and code availability

Due to reasons of confidentiality and user privacy, we cannot share the raw data. However, we can make available all the input distributions and raw empirical results upon request for purposes of reproducibility.

The code used for all experiments is available at: github.com/computationalprivacy/scaling-unicity.

### The unicity model in detail

We propose a simple statistical model M taking into account three population-wide distributions: activity ($P_{A}$), circadian ($P_{C}$), and frequency ($P_{F}$). This model samples location traces for each user independent of other users to estimate unicity of a dataset of size *N*. These location traces are then grouped together to compute unicity.

Formally, the model M can be written as:

(Equation 3) $$M\left(P_{A},P_{C},P_{F},L,N\right)=D=\left(D_{1},\ldots ,D_{N}\right)\text{.}$$

Each $D_{i}\in D$ is a location trace for a unique user, represented as a list of $L_{i}$ records ${\left(X_{i}^{\left(j\right)},C_{i}^{\left(j\right)}\right)}_{j=1}^{L_{i}}$. The length $L_{i}$ of trace $D_{i}$ is sampled from the empirical activity distribution $P_{A}$:

(Equation 4) $$P\left[L_{i}=\ell \right]=P_{A}\left(\ell \right)\text{.}$$

The timestamps of each record in a trace, ${\left(C_{i}^{\left(j\right)}\right)}_{j=1}^{L_{i}}$, are sampled independent of the empirical circadian distribution $P_{C}$:

(Equation 5) $$P\left[C_{i}^{\left(j\right)}=c\right]=P_{C}\left(c\right)\;\forall j\in \left\{1,\ldots ,L_{i}\right\}\text{.}$$

For the spatial component, for each user, a connected sub-graph $S_{i}$ of size 10 is first sampled from the Delaunay tessellation of the antenna coordinates L. This sub-graph is then randomly ordered as a list, which we denote by $S_{i}={\left(S_{i}^{\left(k\right)}\right)}_{k=1}^{10}$ with a slight abuse of notations. Finally, the locations of the records $X_{i}^{\left(j\right)}\in X_{i}$ are sampled independent of $S_{i}$ according to the empirical frequency distribution $P_{F}$:

(Equation 6) $$P\left[X_{i}^{\left(j\right)}=S_{i}^{\left(k\right)}\right]=P_{F}\left(k\right)\;\forall j\in \left\{1,\ldots ,L_{i}\right\}\text{.}$$

Note that when the size of the dataset *N* sampled by our model M increases, this corresponds to sampling more individuals from the same underlying geography. This is what we mean throughout this work when we increase the size of the dataset, e.g., in unicity curves (Figure 1): we consider the dataset to be a growing sample from the same underlying population.

## Supplemental information

Document S1. Supplemental experimental procedures, Figures S1–S5, and Tables S1–S3

Document S2. Article plus Supplemental information

## Footnotes

Supplemental information can be found online at https://doi.org/10.1016/j.patter.2021.100204.
