# Supplementary material for: The risk of re-identification remains high even in country-scale location datasets
Source: Patterns (N Y). 2021 Mar 12;2(3):100204. doi: 10.1016/j.patter.2021.100204 (PMC7961185; doi:10.1016/j.patter.2021.100204)
Supplement: Document S1. Supplemental experimental procedures, Figures S1–S5, and Tables S1–S3 [file mmc1.pdf]

Patterns, Volume *2*

Supplemental information

The risk of re-identification remains high even in country-scale location datasets

Ali Farzanehfar, Florimond Houssiau, and Yves-Alexandre de Montjoye

Supplemental Experimental Procedures for:
The risk of re-identification remains high even in country-scale location datasets

1 Estimated unicity of larger datasets

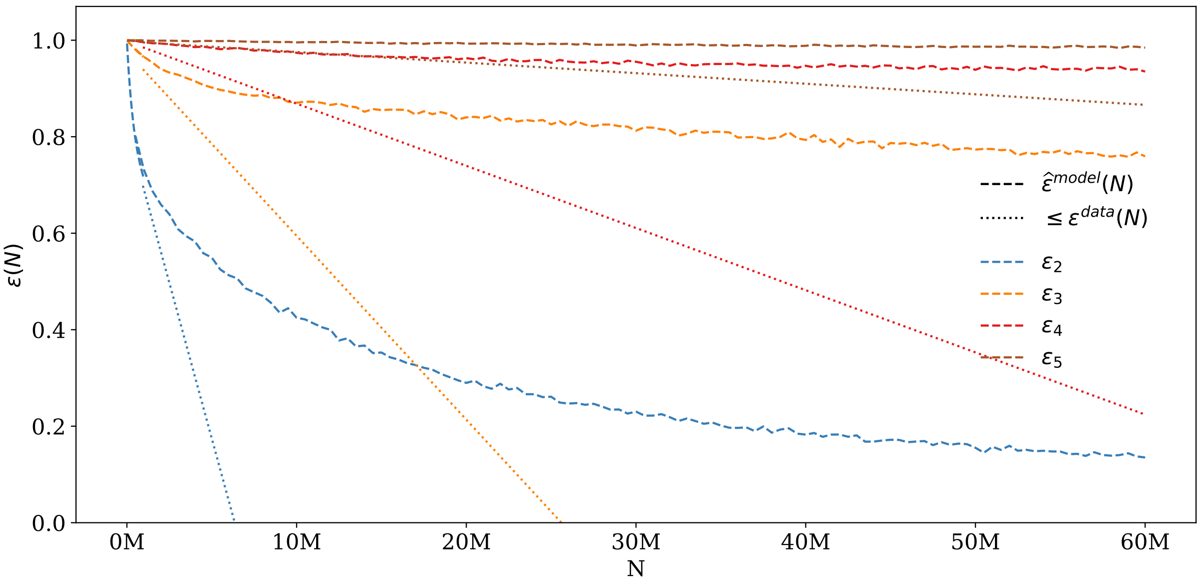

Fig S1 Estimated unicity values along a lower bound on unicity. The estimated unicity remains high even in large dataset. This is confirmed by the lower bound results.

Based on Figure S1, 93% of people would be uniquely identified in a dataset of 60M people using 4 points of auxiliary information with a lower bound at 22%. This bound increases to 87% when 5 points are available. These results re-enforce the conclusion of the main text that the risk of re-identification remains high even in country-scale location datasets.

2 Sensitivity analysis

The unicity model vary with both the frequency distribution (P_F), a power law, and the activity distribution (P_A), a Beta distribution. Broadly speaking, one can observe that:

- **Frequency distribution.** With increasing values of the exponent of the power law unicity decreases

- **Activity distribution.** Given a fitted Beta function $\beta(a, b)$:
  1. Unicity decreases with increasing values of a
  2. Unicity increases with increasing values of b

We here provide more details on the sensitivity analysis conducted in this work. In Figure 2 in the main text we saw that the empirical frequency and activity distributions are each approximated by a power-law and a beta function respectively. These are:

$$\overline{P_F}(k) = \frac{c}{k^\gamma} \tag{S1}$$
$$\overline{P_A}(l) = c \cdot l^{a-1} \cdot (1-l)^{b-1} \tag{S2}$$

Here, we perturb these distributions around their empirical fit. For each of the two, we change their fit parameters to obtain a set of new distributions $P_{\text{q}} \in \mathcal{H}_q^{all}$ where $q \in \{A, F\}$ corresponds to either the frequency, F, or activity, A, distributions. $\mathcal{H}_q^{all}$ corresponds to the set of all possible distributions. The P_q included in this sensitivity analysis are picked such that the earth movers distance (also called first Wasserstein distance) (d_{emd}) between P_q and the observed distribution P_{obs}, is no more than 70% of its maximum distance given. While in general the earth movers distance between two probability distributions is not bounded, in our case, the distributions are defined over a discrete finite space and the distance is thus bounded by a finite number.

The set of distributions included in this analysis are a subset of the elements of $\mathcal{H}_q$ which is constructed as a random sample of $\mathcal{H}_q^{all}$. Therefore, in this analysis, we choose those $P_q \in \mathcal{H}_q \subset \mathcal{H}_q^{all}$ such that:

$$\mathcal{H}_q^{selected} = \left\{P_q \in \mathcal{H}_q \mid d_{emd}(P_q, P_{obs}) \leq 0.7 \cdot \sup_{P_q \in \mathcal{H}_q} \{d_{emd}(P_q, P_{obs})\}\right\} \tag{S3}$$

Our estimate of the parameter values that satisfy this heuristic are included in table S1 and table S2. The constant values c are normalising factors and are only included here for the purposes of reproducibility. The resulting distributions are visible in Figure 3 in the main text.

Each combination of $\overline{P_A}$ and $\overline{P_F}$ yields a new instantiation of the model, generating 4 new sets of unicity estimates — unicity computed with 2, 3, 4 and, 5 points of auxiliary information. The plots in Figure S2 demonstrate that the reduction of unicity in each of these is very similar in form across all the different instantiations. The summary of these results is visible in table S3.

Table S1 $\overline{P_F}$ parameter values

γ	c
0.390	0.173
0.802	0.281
1.214	0.409
1.626	0.540
2.038	0.655
2.450	0.747
2.862	0.816

Table S2 $\overline{P_A}$ parameter values

a	b	c
1.186	5.142	0.0036
1.186	17.094	0.0146
1.186	29.046	0.0275
1.689	5.142	0.009
1.689	17.094	0.0639
1.689	29.046	0.1546
2.193	5.142	0.01927
2.193	17.094	0.2298
2.193	29.046	0.7137

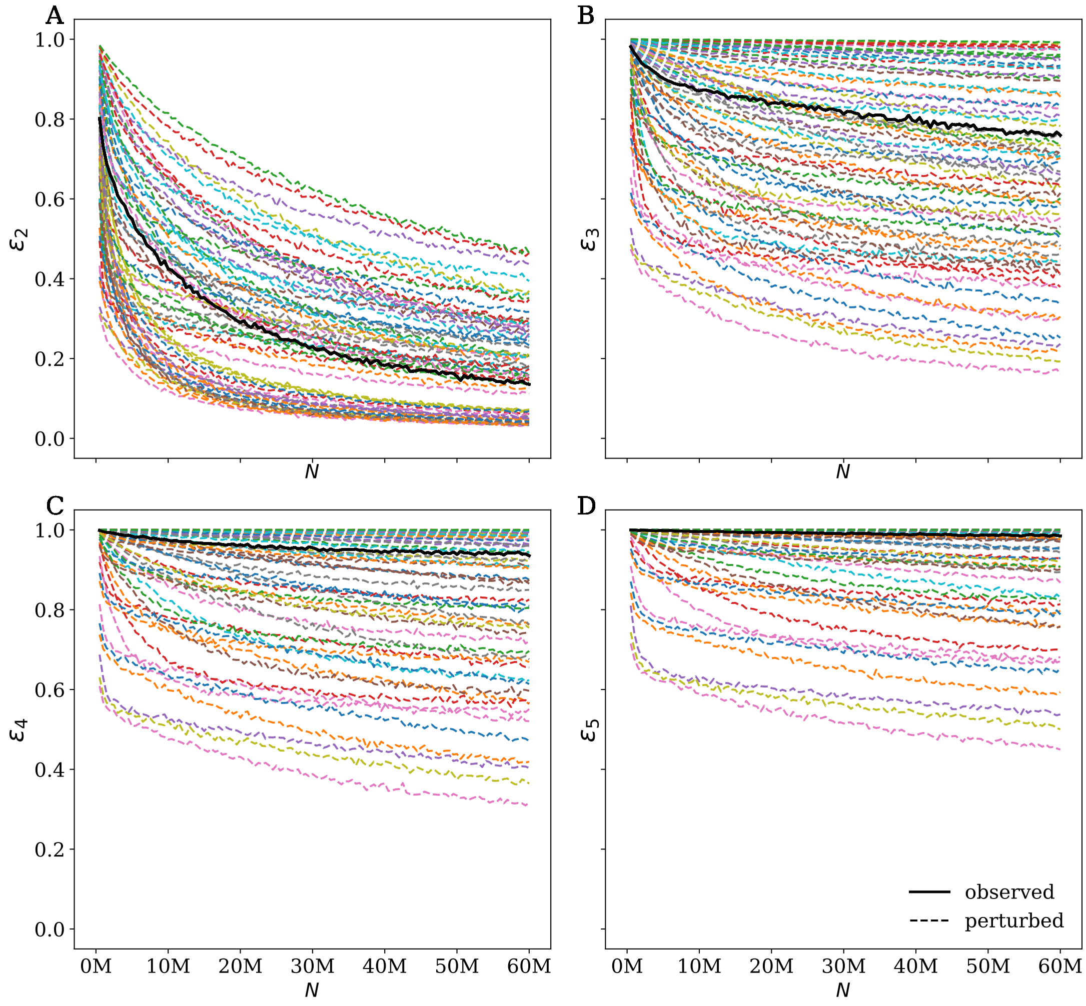

Fig S2 Unicity of trajectories generated using the 63 different perturbed input distributions (dashed) for ε_2 (**A**), ε_3 (**B**), ε_4 (**C**) and, ε_5 (**D**). The solid black line is the unicity model calibrated with the non-perturbed observed input distributions.

	ε_2	ε_3	ε_4	ε_5
Mean	0.182	0.656	0.83	0.908
Standard deviation	0.121	0.24	0.192	0.129
Minimum	0.033	0.162	0.317	0.449
Maximum	0.464	0.991	1	1

Table S3 Summary of unicity results at $N = 60M$ as per the sensitivity analysis.

3 Data set

Our dataset consists of the location traces of close to 1M individuals who have visited at least 10 distinct locations over 3 months for one telecommunication operator. Each record in the data consists of a timestamp with hourly granularity, the GPS coordinates of the antenna the user was connected to the most during the hour, and a unique pseudonym. The records are generated when an individual interacts with the mobile phone network (i.e. makes/receives a call or sends/receives a text). Further, the data contains nearly 6000 antennas that span the whole of the country. Antennas in our database are distributed throughout the country that has quite a heterogeneous geography. The median area covered by an antenna is 16 km^2 with smaller antennas covering areas closer to 0.05 km^2. More than 50% of antennas cover areas that are less than 10 km^2 and the distribution of subscribers across the country roughly follows its population density. Finally, for each trajectory in the data, multiple antennas can be associated to one hour (e.g. multiple calls made within the same hour). In such a case, we pick the most popular antenna within the hour with ties broken randomly.

4 Estimating unicity

Computing the exact unicity of a data set D is extremely computationally expensive (see equation S5). In line with previous work, we estimate unicity by randomly sampling a smaller data set of users $Q \subset D$ and computing the fraction of trajectories in Q that are unique in D given p randomly selected points from each user in Q. Namely, from each trajectory $T^{(i)} \in Q$, p points are picked uniformly at random, labelled $A_p^{(i)} \subset T^{(i)}$. The unicity estimate ε_p is then the fraction of trajectories in Q that are unique in the entire data set D, given $A_p^{(i)}$ as auxiliary information (see algorithm S2 of reference [36] of the main text).

5 Algorithm for graph sub-sampling

One of the four components of the unicity model is a set of 10 distinct antennas S_i visited by the trajectory i (Figure 2D). This location set is obtained following the procedure outlined below:

1. Use the geographic coordinates of all antennas in the location set $\mathcal{L}$ to generate the Delaunay tessellation $\mathcal{G}$ (Full diamonds in Figure 2D of the main text).

2. Pick a starting antenna $S_i^{(j)}$ uniformly at random from $\mathcal{L}$.

3. Add $S_i^{(j)}$ to the set $S_i = \{S_i^{(j)}\}$ (Full red circles in Figure 2D of the main text).

4. Find all the neighbouring antennas to $S_i^{(j)}$ according to $\mathcal{G}$, denoted by $Neighbours(G, j)$.

5. Add all the locations in $Neighbours(G, j)$ to a choice set U_i (Hollow circles in Figure 2D of the main text).

6. Pick, uniformly at random, an antenna $S_i^{(k)}$ from $W_i = U_i \setminus S_i$, and add it to S_i.

7. Repeat steps 3 - 6 until $|S_i| = 10$.

This process effectively samples a connected sub-graph denoted by nodes S_i from the Delaunay tessellation G. This sub-graph will then be used as the set of locations visited by the user i in the unicity model $\mathcal{M}$.

Note that the above algorithm describes the procedure through which the location set S_i is produced. It does not imply that this is how users actually move from node to node.

6 Convexity of unicity

Our linear lower bound on unicity relies on the relationship between unicity and the size of the dataset to be convex. Here, we prove that as long as trajectories are independent and identically distributed, the expected unicity of a population, as a function of population size, follows such a form.

Let $\mathcal{T}$ the set of all possible trajectories. We assume $\mathcal{T}$ to be finite, an assumption which agrees with our empirical setting as the number of possible points (number of hour-location combinations) that could constitute a trajectory is finite, as is the set of all points. We define a data set D as a collection of N trajectories: $D = (D_1, \ldots, D_N),\ D_i \subseteq \mathcal{T}$. A unicity model $\mathcal{M}$ is a randomised mechanism that for a value of N generates a data set D of size N given that:

1. All trajectories $D_i \in D$ are sampled from the *same* distribution T_X

2. All trajectories are sampled *independently* from T_X, meaning $D_i \perp\!\!\!\perp D_j$

The first assumption states that an underlying distribution for population trajectories T_X exists, and it captures correlation between individuals on a large-scale (e.g. commuting patterns, cities,

weekends). The second assumption assumes that the correlation between specific individuals is negligible when estimating unicity.

We can thus write that a data set is a random variable $D = (D_1, \dots, D_N)$, with $D_i \sim T_X$ and $D_i \perp\!\!\!\perp D_j$ if $i \neq j$. In the following notation, all random variables are represented by upper-case letters[1] and their realisations or possible values by lower-case letters[2].

Unicity is computed with knowledge of a random set of points in a trajectory. Given a set $A_p^{d_i} \subseteq d_i$ of p points *picked uniformly at random* from a trajectory d_i, we denote the probability that this trajectory $d_i \in d$ is unique in d as:

$$\mathbb{P}_{A_p^{d_i}} \left[A_p^{d_i} \not\subseteq d_j : \forall j \neq i \right] \tag{S4}$$

Unicity $\varepsilon_p(d)$ for a data set d is then defined as this probability (equation S4) averaged over all trajectories in a particular data set d. Let $N = |d|$, we thus have:

$$\varepsilon_p(d) = \frac{1}{N} \sum_{i=1}^{N} \mathbb{P}_{A_p^{d_i}} \left[A_p^{d_i} \not\subseteq d_j : \forall j \neq i \right] \tag{S5}$$

We are interested in the *expected unicity* of a data set D generated by our randomised mechanism $\mathcal{M}$. This is the expectation of equation S5 taken over all possible data sets d.

$$\bar{\varepsilon}_p(N) = \mathbb{E}_D \left[\varepsilon_p(D) \right] \tag{S6}$$

From here we can use equations S4 and S5 to obtain:

[1] $D_i, X \dots$
[2] $d_i, x \dots$

$$\bar{\varepsilon}_p(N) = \mathbb{E}_D\left[\varepsilon_p(D)\right] \tag{S7}$$

$$= \mathbb{E}_D\left[\frac{1}{N}\sum_{i=1}^{N}\mathbb{P}_{A_p^{D_i}}\left[A_p^{D_i} \not\subseteq D_j : \forall j \neq i\right]\right] \tag{S8}$$

$$= \frac{1}{N}\sum_{i=1}^{N}\mathbb{E}_D\left[\mathbb{P}_{A_p^{D_i}}\left[A_p^{D_i} \not\subseteq D_j : \forall j \neq i\right]\right] \tag{S9}$$

where in equation S9 we have substituted equation S4 while also specifying that the trajectories are now random variables as the expectation is taken over the data set D. Next we will expand this to obtain:

$$\bar{\varepsilon}_p(N) = \frac{1}{N}\sum_{i=1}^{N}\mathbb{E}_D\left[\mathbb{P}_{A_p^{D_i}}\left[A_p^{D_i} \not\subseteq D_j : \forall j \neq i\right]\right] \tag{S10}$$

$$= \frac{1}{N}\sum_{i=1}^{N}\sum_{d\in\mathcal{T}^N}\mathbb{P}[D=d]\cdot\mathbb{P}_{A_p^{d_i}}\left[A_p^{d_i} \not\subseteq d_j : \forall j \neq i\right] \tag{S11}$$

In equation S11 we used the definition of the expected value to obtain the sum over all possible data sets d. Let us expand this term (representing the other factor by a $\circ$):

$$\sum_{d\in\mathcal{T}^N}\mathbb{P}[D=d]\circ = \sum_{d\in\mathcal{T}^N}\mathbb{P}[D=(d_1,d_2,\ldots,d_N)]\circ \tag{S12}$$

$$= \sum_{d\in\mathcal{T}^N}\prod_{k=1}^{N}T_X(d_k)\circ \tag{S13}$$

$$= \sum_{d_1\in\mathcal{T}}\cdots\sum_{d_N\in\mathcal{T}}\prod_{k=1}^{N}T_X(d_k)\circ \tag{S14}$$

$$= \sum_{d_i\in\mathcal{T}}T_X(d_i)\sum_{d_1\in\mathcal{T}}\cdots\sum_{d_{i-1}\in\mathcal{T}}\sum_{d_{i+1}\in\mathcal{T}}\cdots\sum_{d_N\in\mathcal{T}}\prod_{k\neq i}T_X(d_k)\circ \tag{S15}$$

Going from S14 to S15, the ith component of the sum over the trajectories was pulled out to the front for later use. Going back to S9 we obtain:

$$\bar{\varepsilon}_p(N) = \frac{1}{N}\sum_{i=1}^{N}\sum_{d\in\mathcal{T}^N}\mathbb{P}[D=d]\,\mathbb{P}_{A_p^{d_i}}\left[A_p^{d_i}\not\subseteq d_j : \forall j\neq i\right] \tag{S16}$$

$$= \frac{1}{N}\sum_{i=1}^{N}\sum_{d_i\in\mathcal{T}} T_X(d_i)\sum_{d_1\in\mathcal{T}}\cdots\sum_{d_{i-1}\in\mathcal{T}}\sum_{d_{i+1}\in\mathcal{T}}\cdots\sum_{d_N\in\mathcal{T}}\prod_{k\neq i}T_X(d_k)\mathbb{P}_{A_p^{d_i}}\left[A_p^{d_i}\not\subseteq d_j : \forall j\neq i\right] \tag{S17}$$

At this stage equation S17 contains the expression for the expected value over $D\setminus\{D_i\}$ of the probability. We consolidate all sums to reflect this fact:

$$\bar{\varepsilon}_p(N) = \frac{1}{N}\sum_{i=1}^{N}\sum_{d_i\in\mathcal{T}} T_X(d_i)\sum_{d_1\in\mathcal{T}}\cdots\sum_{d_{i-1}\in\mathcal{T}}\sum_{d_{i+1}\in\mathcal{T}}\cdots\sum_{d_N\in\mathcal{T}}\prod_{k\neq i}T_X(d_k)\mathbb{P}_{A_p^{d_i}}\left[A_p^{d_i}\not\subseteq d_j : \forall j\neq i\right] \tag{S18}$$

$$= \frac{1}{N}\sum_{i=1}^{N}\sum_{d_i\in\mathcal{T}} T_X(d_i)\mathbb{E}_{D\setminus\{D_i\}}\left[\mathbb{P}_{A_p^{d_i}}\left[A_p^{d_i}\not\subseteq D_j : \forall j\neq i\right]\right] \tag{S19}$$

The probability expression essentially represents the chances that any randomly picked p points from d_i, appear in any other trajectory d_j. This can be computed as, by developing the probability over $A_p^{d_i}$:

$$\mathbb{P}_{A_p^{d_i}}\left[A_p^{d_i}\not\subseteq D_j : \forall j\neq i\right] = \sum_{a_p^{d_i}}\frac{1}{C_{|d_i|}^{p}}\mathbb{1}_{D_{j\neq i}}\left[a_p^{d_i}\not\subseteq D_j,\ \forall j\neq i\right] \tag{S20}$$

Where $C_{|d_i|}^{p}$ is the number of ways p points can be picked from the trajectory d_i. Inserting this back into equation S19 results in the following.

$$\bar{\varepsilon}_p(N) = \frac{1}{N}\sum_{i=1}^{N}\sum_{d_i\in\mathcal{T}} T_X(d_i)\mathbb{E}_{D\setminus\{D_i\}}\left[\sum_{a_p^{d_i}}\frac{1}{C_{|d_i|}^{p}}\mathbb{1}_{D_{j\neq i}}\left[a_p^{d_i}\not\subseteq D_j,\ \forall j\neq i\right]\right] \tag{S21}$$

Note that: 1) The expression above is agnostic to the index i which cancels with the leading factor of $\frac{1}{N}$ and, 2) The expected value of the indicator variable of a random event is the probability

of that event. Noting that $D \setminus \{D_i\}$ represents the same variable set as the ones impacting the indicator function $D_{j\neq i}$, we move in the expectation operator past the sum and the constant factor and therefore re-write equation S21 as:

$$\bar{\varepsilon}_p(N) = \sum_{d_i \in \mathcal{T}} T_X(d_i) \sum_{a_p^{d_i}} \frac{1}{C_{|d_i|}^p} \mathbb{P}_{D_{j\neq i}}\left[a_p^{d_i} \not\subseteq D_j\right] \tag{S22}$$

$$= \sum_{d_i \in \mathcal{T}} T_X(d_i) \sum_{a_p^{d_i}} \frac{1}{C_{|d_i|}^p} \prod_{j\neq i} \mathbb{P}_{D_j}\left[a_p^{d_i} \not\subseteq D_j\right] \tag{S23}$$

$$= \sum_{d_i \in \mathcal{T}} T_X(d_i) \sum_{a_p^{d_i}} \frac{1}{C_{|d_i|}^p} \prod_{j\neq i} \mathbb{P}_X\left[a_p^{d_i} \not\subseteq X\right] \tag{S24}$$

$$= \sum_{d_i \in \mathcal{T}} T_X(d_i) \sum_{a_p^{d_i}} \frac{1}{C_{|d_i|}^p} \left(\mathbb{P}_X\left[a_p^{d_i} \not\subseteq X\right]\right)^{N-1} \tag{S25}$$

In equation S23 we used the independence of different trajectories, and we used the fact that all trajectories are identically distributed in going from equation S24 to S25. Finally, with a change of dummy variables from d_i to y we obtain:

$$\bar{\varepsilon}_p(N) = \sum_{y \in \mathcal{T}} \frac{1}{C_{|y|}^p} T_X(y) \sum_{a_p^y} \left(\mathbb{P}_X\left[a_p^y \not\subseteq X\right]\right)^{N-1} \tag{S26}$$

Finally we note that the form of equation S26 is:

$$\bar{\varepsilon}_p(N) = \sum_{y \in \mathcal{T}} \frac{1}{C_{|y|}^p} T_X(y) \sum_{a_p^y} \left(\mathbb{P}_X\left[a_p^y \not\subseteq X\right]\right)^{N-1} \tag{S27}$$

$$= \sum_{y \in \mathcal{T}} \frac{1}{C_{|y|}^p} T_X(y) \sum_{x(y)} \left(f(x)\right)^{N-1} \tag{S28}$$

$$= \sum_{y \in \mathcal{T}} \frac{1}{C_{|y|}^p} T_X(y) g(y, N) \tag{S29}$$

We now show that $\bar{\varepsilon}_p(N)$ is a convex function of a real-valued N. From this, we obtain that the series $(\bar{\varepsilon}_p(N))_{N=1,2,...}$ is a convex sequence (this is a direct consequence of the definition of convexity), which concludes the proof.

Functions of the form $f(m) = b^m$ are convex for all positive m and b. Further, the sum of convex functions is itself convex, and therefore $g(y, N)$ is convex in N in equation S29. Using this fact one more time we obtain that $\bar{\varepsilon}_p(N)$ must also be convex, since all elements being multiplied out in equations S27 to S29 are probabilities and are thus non-negative.

Finally, while our analysis considers real-valued functions, a direct consequence of this convexity result is that the sequence $\{\varepsilon(D(N))\}_{N=1,2,3...}$, which represents the population dependence of unicity for discrete population sizes, is also a convex sequence.

Given this result, the unicity of a dataset of size N will always obey the following relation (treating unicity as a function of a real-valued N):

$$\varepsilon(D(N)) \geq \varepsilon(D(N')) + (N - N') \cdot \left.\frac{d\varepsilon}{dN}\right|_{N=N'} \tag{S30}$$

The discrete equivalent of this expression replaces the differentiation operator by a difference operator. Re-arranged, this gives a lower bound for unicity:

$$\varepsilon\big(D(N')\big) - \varepsilon\big(D(N)\big) \leq (N - N') \cdot \Big(\varepsilon\big(D(N'-1)\big) - \varepsilon\big(D(N')\big)\Big) \tag{S31}$$

Unicity being a strictly decreasing convex function, it is lower-bounded by its linear tangent.

7 Estimating uniqueness by subsampling a larger dataset

El Emam [42] proposed a method to estimate the uniqueness of a population-size (N) dataset given its unicity $\varepsilon(m)$ for a smaller population size m: generating a dataset D of size N where a fraction α of records are unique, and all other records are identical to exactly one other record. The parameter α is chosen such that the expected estimated uniqueness on a sample of size m, which we denote by $\nu_D(m)$, is equal to the empirical unicity.

By applying this method to prior results [36, main text] ($m = 10^6$, $\varepsilon_4(m) = 0.9$), El Emam estimates that the real population uniqueness for a population of size $N = 22 \cdot 10^6$ is of about 1%. This is used as a basis to argue that the unicity of a sample strongly overestimates the uniqueness of population-scale data, a claim this paper refutes.

In the remainder of this section, we prove that El Emam's method computes a worst-case lower bound on the uniqueness of a population given a sample.

For this, we first show that for any dataset D of size N, the sample uniqueness as a function of the sample size m, $\nu_D(m)$, is a convex decreasing function of m. We then show that for the dataset created by El Emam's method, D_α, sample uniqueness $\nu_{D_2}(m)$ is an *affine* function of m. Hence, the uniqueness estimated with El Emam's method will be lower or equal to the uniqueness of *any* other dataset for all (with $m' > m$ including for $m' = N$).

Let $D = (d_1, \dots, d_N)$ a population-scale dataset of size $N > 1$, with records d_i in some arbitrary set $\mathcal{D}$. A *matching* relation $\mathcal{M} : D \times D \to \{0, 1\}$ is defined over records in D, that outputs 1 iff two records are identical. This relation defines k equivalence classes $\mathcal{C}_1, \dots, \mathcal{C}_k$ of sizes $n_1, \dots, n_k$, such that $i, j \in \mathcal{C}_l \Leftrightarrow \mathcal{M}(d_i, d_j) = 1$. We show below that this list of class sizes $(n_1, \dots, n_k)$ uniquely characterises the behavior of the uniqueness in a sample of size $m < N$.

A dataset $D' \subset D$ of size $m \leq N$ (the sample dataset) is sampled uniformly without replacement from D. For each record d_i in D, we define the binary random variable $S_i = I\{d_i \in D'\}$. S_i follows a Bernoulli distribution with parameter $\frac{m}{N}$, and all Y_i satisfy the following equation: $\sum_{i=1}^{N} Y_i = m$. From this, we define the *count per class* variable $N_j = \sum_{i \in C_j} S_i$ for $j \in \{1, \dots, k\}$.

Using these notations, the sample uniqueness for a sample size $m \leq N$ is defined as the expected fraction of records of D' that are unique in D', i.e. that are the only records of their class in D':

$$\nu_D(m) = \mathbb{E}\left[\frac{1}{m}\sum_{i=1}^{k} I\{N_i = 1\}\right] = \frac{1}{m}\sum_{i=1}^{k} \mathbb{P}\left[N_i = 1\right]$$

Denote by $\binom{a}{b}$ the number of subsets of size a of a set of size b ("a choose b"). The probability that $N_i = 1$ for some class of size n_i is equal to the fraction of samples of size m from the dataset (of size N) that have exactly 1 record from the class:

$$\mathbb{P}\left[N_i = 1\right] = \frac{\binom{1}{n_i} \cdot \binom{m-1}{N-n_i}}{\binom{m}{N}} = \frac{n_i \cdot \frac{(N-n_i)!}{(m-1)!\,(N-n_i-m+1)!}}{\frac{N!}{m!\,(N-m)!}} = n_i \cdot m \cdot \frac{(N-n_i)!}{N!} \cdot \prod_{j=0}^{n_i-2} (N-m-j)$$

We then use this expression to compute the sample uniqueness:

$$\nu_D(m) = \frac{1}{m}\sum_{i=1}^{k} m\, n_i \frac{(N-n_i)!}{N!} \prod_{j=0}^{n_i-2} (N-m-j) = \sum_{i=1}^{k} n_i \frac{(N-n_i)!}{N!} \cdot \prod_{j=0}^{n_i-2} (N-m-j)$$

We show that ν_D is a convex function of m, for $m \in [1, N]$, for any dataset D of size N. For this, observe that $\nu_D(m)$ is a linear combination of the convex function $\hat{f}_{n_i}(m)$ as defined in Lemma 7.1, for $i = 1, \dots, k$, with nonnegative linear coefficients. We show in Lemma 7.1 that $\hat{f}_{n_i}$ is a convex

function of m for all n_i, and thus, $\nu_D(m)$ is a convex, decreasing, function of m.

Lemma 7.1 Let $n_i \in \mathbb{N}_0$, $N > n_i$. The function $\hat{f}_{n_i}$ defined as:

$$\hat{f}_{n_i} : [0, N] \to \mathbb{R} : m \mapsto \begin{cases} \prod_{j=0}^{n_i-2}(N-m-j) & \text{if } m \leq N - n_i + 2 \\ 0 & \text{if } m > N - n_i + 2 \end{cases}$$

is a convex, decreasing, non-negative function of m.

Proof. First, Define the function $f_{n_i} : \mathbb{R}^+ \to \mathbb{R} : m \mapsto \prod_{j=0}^{n_i-2}(N-m-j)$. We show that $\forall\, m$ such that $N \geq m + n_i - 2$, the first derivative is non-positive, and the second derivative of f_{n_i} is non-negative:

$$\begin{cases} \frac{\mathrm{d}f_{ni}}{\mathrm{d}m}(m) = (-1) \cdot \sum_{l=0}^{n_i-2} \prod_{j \neq l}(N-m-j) \leq 0 \\ \frac{\mathrm{d}^2 f_{ni}}{\mathrm{d}m^2}(m) = \sum_{l=0}^{n_i-2} \sum_{j \neq l} \prod_{s \neq j, s \neq l}(N-m-s) \geq 0 \end{cases}$$

and thus f_{n_i} is decreasing and convex over $[0, m + n_i - 2]$. From this, one gets that $\hat{f}_{n_i}$ is also a decreasing non-negative function over $[0, N]$. Further, since $f(N - n_i + 2) = 0$, this function is continuous.

We now show that $\hat{f}_{n_i}$ satisfies the definition of convexity over $[0, N]$: $\forall x, y \in \mathbb{R}^+$ with $x \leq y$, and $\forall \lambda \in [0,1]$, $\hat{f}_{n_i}(\lambda x + (1-\lambda)y) \leq \lambda x + (1-\lambda)y$. Denote $z = \lambda x + (1-\lambda)y$, and $\kappa = N - n_i + 2$, and observe that:

- Either $x \leq y \leq \kappa$, and the inequality holds by convexity of f_{n_i} over $[0, \kappa]$;
- Either $\kappa \leq x \leq y$, and the inequality holds since $\hat{f}_{n_i}(x) = \hat{f}_{n_i}(z) = \hat{f}_{n_i}(y) = 0$.
- Either $x < \kappa \leq z \leq y$, and the inequality holds since $\hat{f}_{n_i}(x) > 0$ and $\hat{f}_{n_i}(y) = \hat{f}_{n_i}(z) > 0$.
- Either $x \leq z < \kappa \leq y$. Then, there exists $\mu \in [0,1]$ such that $z = \mu x + (1-\mu)\kappa$. By convexity of f_{n_i}, we have $\hat{f}_{n_i}(z) \leq \mu \hat{f}_{n_i}(x) + (1-\mu) f_{n_i}(\kappa) = \mu \hat{f}_{n_i}(x)$. Further, observe that $z = \mu x + (1-\mu)\kappa = \lambda x + (1-\lambda)y \geq \lambda x + (1-\lambda)\kappa$, and thus $\lambda \geq \mu$. Combined together, this implies $\hat{f}_{n_i}(z) \leq \mu \hat{f}_{n_i}(x) \leq \lambda \hat{f}_{n_i}(x) = \lambda \hat{f}_{n_i}(x) + (1-\lambda)\hat{f}_{n_i}(y)$, since $\hat{f}_{n_i}(x) > 0$ and $\hat{f}_{n_i}(y) = 0$.

□

We then compute the sample uniqueness of the dataset used by El Emam's method, which we call D_α. By construction, this dataset has $\alpha N \in \mathbb{N}$ classes of size $n_i = 1,\ i = 1, \ldots, \alpha N$, and $\frac{(1-\alpha)N}{2}$ classes of size $n_i = 2,\ i = \alpha N + 1, \ldots, k$. We thus get:

$$\nu_{D_\alpha}(m) = \alpha N \cdot \frac{1}{N} + \frac{(1-\alpha)N}{2} \cdot 2\frac{N-m}{N(N-1)} = \alpha + (1-\alpha)\left(1 - \frac{m-1}{N-1}\right)$$

which is an affine function of m.

Let (m^*, ε^*) an empirical measurement of uniqueness on a dataset of size m^*, assumed to be a uniform sample from a sample of size N. We show that if there exists an α for which $\nu_{D_\alpha}(m^*) = \varepsilon^*$, then *any* other dataset D of size N such that $\nu_D(m^*) = \varepsilon^*$ will have higher sample uniqueness for any $m' \geq m^*$: $\nu_D(m') \geq \nu_{D_\alpha}(m')$. This is a direct consequence of the convexity of $\nu_D(m)$ and linearity of $\nu_{D_\alpha}(m)$. Indeed, convexity for $\nu_D(m)$ implies that, $\forall m' \geq m^*$ (using the definition with $x = m'$, $y = 1$ and $\lambda' = \frac{m^*-1}{m'-1} \leq 1$):

$$\varepsilon^* = \nu_D(m^*) \leq \lambda' \cdot \nu_D(m') + (1 - \lambda') \cdot \nu_D(1)$$

which in turn entails, since $\nu_D(1) = 1$:

$$\nu_D(m') \geq \frac{\varepsilon^* - 1 + \lambda'}{\lambda'} = \frac{m' - 1}{m^* - 1}(\varepsilon^* - 1) + 1$$

Observe that choosing $\alpha^* = 1 + \frac{N-1}{m^*-1}(\varepsilon^* - 1)$ in El Emam's method, if $\alpha \leq 1$, is such that $\nu_{D_{\alpha^*}}(m^*) = \varepsilon^*$. If $\alpha > 1$, then El Emam's method cannot be used. We then get, for all $m' \geq m^*$:

$$\nu_{D_{\alpha^*}}(m') = \frac{m' - 1}{m^* - 1}(\varepsilon^* - 1) + 1$$

And thus, for *any* dataset D of size N, if α exists, we have that, $\forall\, m' \geq m^*$:

$$\nu_{D_{\alpha^*}}(m') \leq \nu_D(m')$$

8 A naive unicity model: the combinatorial approach

A naive approach to model unicity is to approach it as a purely combinatorial problem with every time and location pair being equally likely to appear in a trace.

Given N trajectories of length λ hours, the model assigns to each of the λ hours a particular location $\mathcal{L}_i$ from the $|\mathcal{L}|$ possible locations uniformly at random.

Given p randomly picked points $A_p^{D_i}$ from a trajectory D_i, the probability of D_i being uniquely identifiable given $A_p^{D_i}$ is equal to the probability of all other $N - 1$ trajectories not containing all of $A_p^{D_i}$:

$$\mathbb{P}\left[A_p^{D_i} \not\subseteq D_j \; \forall\, j \neq i\right] = \prod_{i \neq j}^{N} \left(1 - \mathbb{P}[A_p^{D_i} \subseteq D_j]\right) \tag{S32}$$

The probability $P[A_p^{D_i} \subseteq D_j]$ can be computed using the fact that the probability of each of the $|\mathcal{L}|$ locations appearing in $A_p^{(i)}$ is equal:

$$\mathbb{P}\left[A_p^{D_i} \not\subseteq D_j\right] = \prod_k^p \mathbb{P}[\mathcal{L}_k \in A_p^{D_i}] = \left(\frac{1}{|\mathcal{L}|}\right)^p \tag{S33}$$

The definition of our unicity estimate is the average of equation S32 over all trajectories. Since the trajectories are independently generated, this equation indeed measures the unicity of the combinatorial model. Using the expression from equation S33 we then have:

$$\begin{aligned} \varepsilon_p^{comb}(N) &= \prod_{i \neq j}^N \left(1 - \mathbb{P}[A_p^{D_i} \subseteq D_j]\right) \\ &= \prod_{i \neq j}^N \left(1 - \frac{1}{|\mathcal{L}|^p}\right) \\ &= \left(1 - \frac{1}{|\mathcal{L}|^p}\right)^{N-1} \end{aligned} \tag{S34}$$

where equation S34 gives a simple form for the naive model.

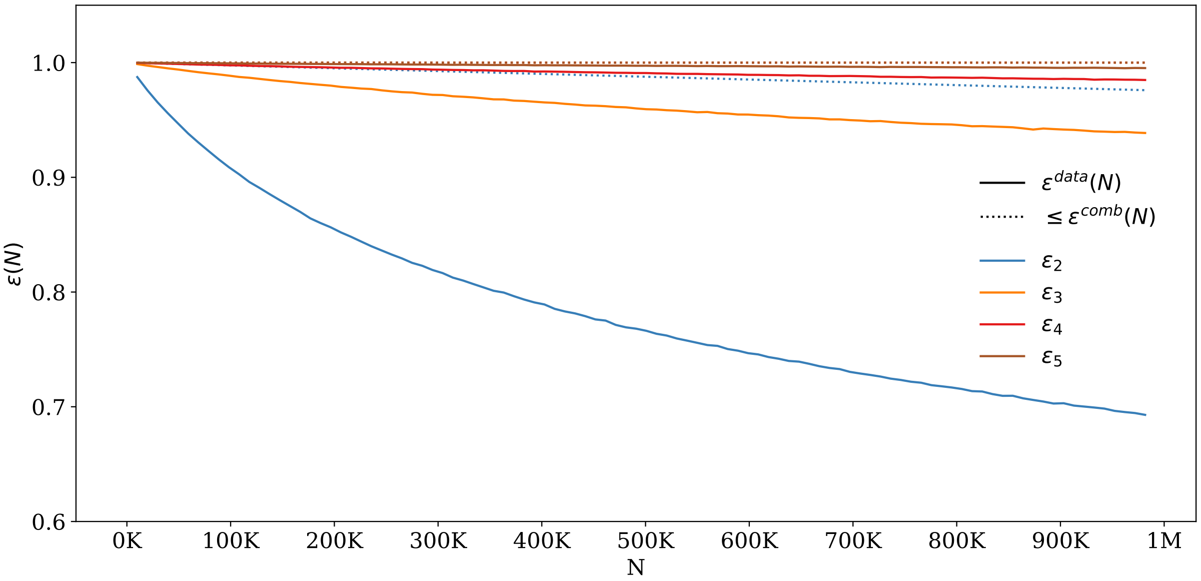

Fig S3 The combinatorial unicity model (dotted lines) greatly overestimates empirical unicity (solid lines).

The combinatorial unicity model estimates are compared to empirical unicity in Figure S3. It is clear that the combinatorial model greatly overestimates unicity values with the dotted lines hugging the region of $\varepsilon \approx 1$ for nearly all values of p.

9 Dependence of the unicity model on input distributions

For an expanded discussion see the main text.

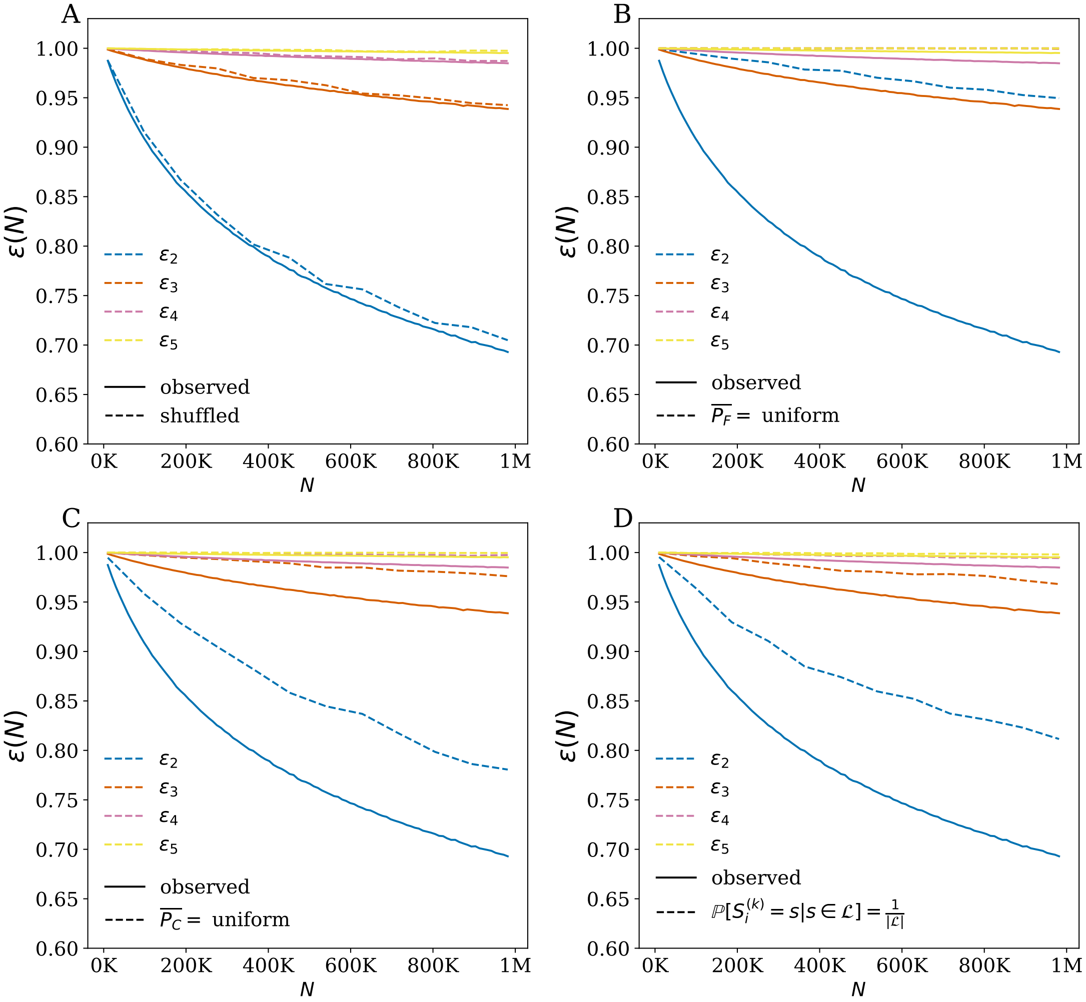

Fig S4 Unicity of alternative models compared to observed unicity. **A:** The space and time components of trajectories are shuffled. The P_F distribution (**B**) and the P_C distribution (**C**) are separately replaced with the uniform distribution. **D:** The antenna set S_i is populated with uniformly picked antennas from $\mathcal{L}$ at random, instead of using the sub-graph sampling method.

10 Convergence of the unicity model

The unicity model extracts a set of 3 distributions (collectively called R_n when computed from data of size (n)) to model the decrease of unicity. We now show how estimates of unicity converge as a function of the size of the data set n from which the distributions are extracted with the following approach:

1. We instantiate the unicity model using input distributions R_n with increasing n as sampled uniformly from the data set of 1M trajectories
2. We use each instantiation to obtain unicity values for data sets ranging from 10K to 1M trajectories. This gives a vector of unicity values
3. Finally, we compare each of these unicity vectors, to the vector obtained by a model instantiated using the entire data (i.e. $n = 1M$) using the Root Mean Squared Error of the vectors.

Figure S5 shows that for varying values of n (the horizontal axis), the instantiations of the unicity model quickly converge to the model with $n = 1M$. Indeed, the rate at which the input distributions converge is such that even with only $n = 2.5K$ ($\approx$ $\%0.0025$ of the data) the results of the unicity models are largely the same as a unicity model that used all 1M trajectories as its input (stable RMSE values). Finally, lines of best fit are also included showing almost horizontal lines across the board.

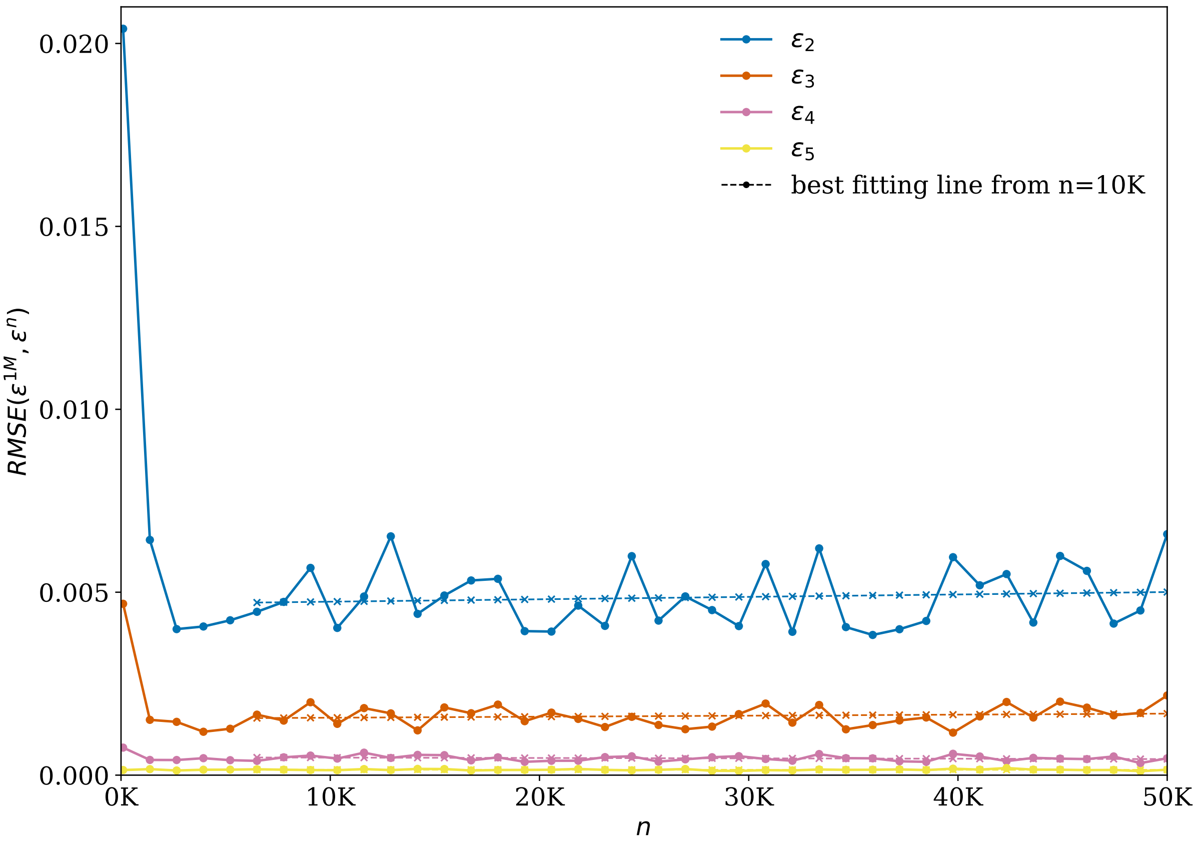

Fig S5 Convergence of the unicity model as the size of the sample (n) from which the input distributions are extracted increases. In solid, the RMSE (vertical axis) of unicity models instantiated with distributions extracted from varying number of trajectories (horizontal axis) is displayed. The dotted line is the best fitting line to each learning curve starting from n=10,000.
