# Supplementary material for: The risk of re-identification remains high even in country-scale location datasets
Source: Patterns (N Y). 2021 Mar 12;2(3):100204. doi: 10.1016/j.patter.2021.100204 (PMC7961185; doi:10.1016/j.patter.2021.100204)
Supplement: Document S2. Article plus Supplemental information [file mmc2.pdf]

Patterns

Article

The risk of re-identification remains high even in country-scale location datasets

Highlights

- Re-identification risk is statistically modeled and shown to decrease slowly with dataset size
- With increasing dataset size, the unicity decrease is lower-bounded and convex
- Previous estimates of unicity unrealistically underestimated the risks
- Individuals are likely re-identifiable in country-size location data and other high-dimensional datasets

Authors

Ali Farzanehfar, Florimond Houssiau, Yves-Alexandre de Montjoye

Correspondence

demontjoye@imperial.ac.uk

In Brief

Researchers have claimed that individuals could not be re-identified in large-scale location datasets, making them safe. We here empirically measure and mathematically model the relationship between the size of a dataset and the risk of re-identification. Our results show that the risk decreases slowly with dataset size, making even large country-scale datasets very likely to be re-identifiable.

Farzanehfar et al., 2021, Patterns *2*, 100204
March 12, 2021 © 2021 The Authors.
https://doi.org/10.1016/j.patter.2021.100204

CellPress

CellPress
OPEN ACCESS

Article

The risk of re-identification remains high even in country-scale location datasets

Ali Farzanehfar,[1] Florimond Houssiau,[1] and Yves-Alexandre de Montjoye[1,2,*]
[1]Department of Computing, Imperial College London, London SW7 2AZ, UK
[2]Lead contact
*Correspondence: demontjoye@imperial.ac.uk
https://doi.org/10.1016/j.patter.2021.100204

THE BIGGER PICTURE Data about us are being collected in many different ways, when we use our bank cards, use our phones, browse the web, or even drive our cars. These datasets contain detailed information about our lives. For each person, a dataset might contain thousands to tens of thousands of records. Previous research has shown that knowing just a few points about a target can single out the vast majority of people in location datasets. However, some had argued the risk of re-identification becomes negligible if we look at large-scale datasets containing tens of millions of people.

Here, we empirically measure, mathematically model, and provide a lower bound on the relationship between the size of a dataset and the risk of re-identification. Our results all show that re-identification risk decreases very slowly with increasing dataset size. Contrary to previous claims, people are thus very likely to be re-identifiable even in country-scale datasets.

1 2 3 4 5 **Proof-of-Concept:** Data science output has been formulated, implemented, and tested for one domain/problem

SUMMARY

Although anonymous data are not considered personal data, recent research has shown how individuals can often be re-identified. Scholars have argued that previous findings apply only to small-scale datasets and that privacy is preserved in large-scale datasets. Using 3 months of location data, we (1) show the risk of re-identification to decrease slowly with dataset size, (2) approximate this decrease with a simple model taking into account three population-wide marginal distributions, and (3) prove that unicity is convex and obtain a linear lower bound. Our estimates show that 93% of people would be uniquely identified in a dataset of 60M people using four points of auxiliary information, with a lower bound at 22%. This lower bound increases to 87% when five points are available. Taken together, our results show how the privacy of individuals is very unlikely to be preserved even in country-scale location datasets.

INTRODUCTION

Throughout our day, we interact with many digital services when using our phone, paying with our credit card, or using public transport with a smart card. This results in our location data being collected broadly, sometimes on the scale of countries. For instance, Vodafone UK collects location trajectories of 20M citizens[1]—a third of the population—while up to 5 million people use London's subway daily.[2]

Location data have been used extensively in research. In urban planning, mobility data can be used to monitor urban activity[3] and help design better cities.[4] In epidemiology, it has been used to monitor and mitigate the spread of infectious diseases such as Ebola and COVID-19.[5–10] In computational social science, it has allowed us to gain unprecedented insights into the spatial distribution of poverty,[11] and even to study the impact of mass employment layoffs on society.[12] Further, the use of location data has withstood scrutiny into potential biases in their collection mechanisms.[13]

Despite this, the large-scale collection and use of location data has raised serious privacy concerns. It consists of fine-grained records of where we are and how we move around, and was considered sensitive by 82% of Americans in a recent survey.[14] Location data can furthermore be used to predict individuals' income,[11,15] their home and work locations,[16–21] when they sleep and wake up,[22–26] their gender and age,[27] their personality,[28] who their friends are,[29,30] and where they tend to socialize.[31]

This is an open access article under the CC BY license (http://creativecommons.org/licenses/by/4.0/).

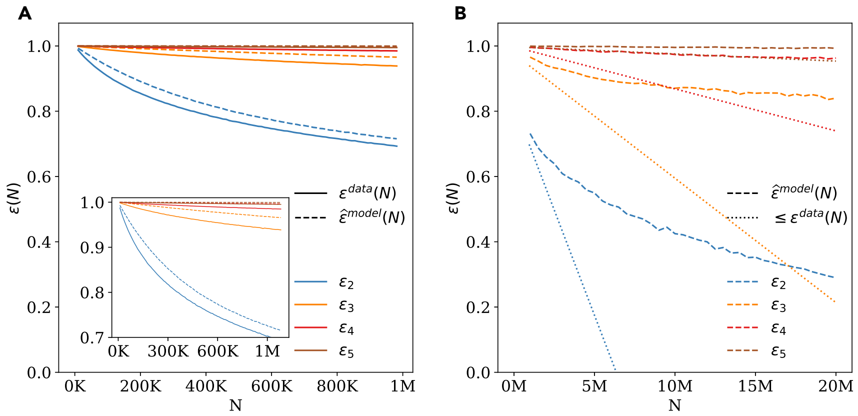

Figure 1. The relationship between unicity and dataset size

(A) Empirical (solid lines) and estimated (dashed lines) unicity decreases slowly with the size of the dataset. Inset: close up of the region $\epsilon \geq 0.7$.

(B) The estimated unicity remains high even in large datasets. This is confirmed by the lower bound results (dotted lines). Taken together, these results strongly suggest that unicity remains high even in country-scale datasets.

Unicity has been proposed as a measure for the risk of re-identification in anonymous datasets and was used to show how four points of auxiliary information (places and times where someone was) are enough to uniquely identify 95% of people in a large-scale location dataset.[32] These four points of auxiliary information could be in the form of geo-tagged "tweets," online check-ins, or information obtained by more traditional means, such as observing someone making a call. Unicity (ϵ_p) is defined as the fraction of trajectories that are unique based on knowledge of p randomly chosen points in a given trajectory. Unicity has since been used to quantify re-identification risk across a number of domains, including the mobility of vehicles,[33] apps downloaded by smartphones over time,[34,35] smart cards used in public transport,[24] credit card transaction histories,[36] and location data from mobile phones in a number of countries.[32,37,38] A range of studies have furthermore exploited the unicity of datasets to re-identify people. Narayanan and Shmatikov famously showed that close to 90% of people could be re-identified in the Netflix dataset,[39] while Riederer and colleagues used the unicity of traces to match the same individual across multiple datasets.[40]

Researchers and industry practitioners have, however, argued that these high unicity numbers are an artifact of the small size of the datasets considered, and are overestimating the risk of re-identification.[41–43] For instance, Riederer et al.[40] relied on a location dataset of 1.7k people, while other case studies report unicity on dataset sizes ranging from several thousands (respectively 12k and 55k)[33,34] to over 1 million people (1.5M).[32] Examining a published study,[36] El Emam et al. estimated that the unicity of a dataset of $\approx$20M trajectories will be as low as 1% given four points of auxiliary information, the conclusion being that privacy was preserved in such large datasets.[42]

We here (1) study 3 months of location data and show empirically that unicity decreases slowly with the size of the dataset, (2) approximate this decrease with a simple statistical model taking into account three population-wide marginal distributions along with the underlying geography, and (3) prove that the decrease in unicity is a convex function of the dataset size and obtain a linear lower bound on unicity. We finally perform a sensitivity analysis suggesting that the decrease in unicity is agnostic to broad perturbations in the input distributions. These results disprove previous claims, instead showing that unicity is likely to remain high even in country-scale datasets.

RESULTS

Our experiments are performed on a dataset of call detail records containing the location of 1M individuals over 3 months. Each record contains a unique user ID, an hourly time stamp, and an antenna ID, which relates to a location (see Supplemental information for more details). We formally model this dataset as a sequence, $D = (D_1, \ldots, D_N)$, populated with user time/location traces of the type $D_i = (X_i, C_i)$. X_i and C_i are lists of positions (antennas) and times (hours) representing the spatial and temporal components of a user's location trace.

Using this dataset, we empirically study the decrease in unicity with the dataset size by randomly sampling individuals from our original dataset and measuring the unicity of the sample as we increase its size (see Experimental procedures for details). We use the formal definition of unicity and the estimation algorithm S2 from de Montjoye et al.[36] In line with previous work, we use the subscript p in $\epsilon_p(N)$ to indicate the number of points of auxiliary information used in the computation of unicity.

Figure 1A shows that unicity empirically decreases slowly with the size of the dataset. With three points of auxiliary information, unicity (solid orange line) goes down from $\epsilon_3(100\text{K}) = 0.98$ in a dataset of 100,000 people to $\epsilon_3(1\text{M}) = 0.93$ in a dataset of a million people. With two points (solid blue line) this decreases slightly faster, reaching $\epsilon_2(1\text{M}) = 0.69$, while unicity with four points or more (solid red and brown lines) decreases very slowly with $\epsilon_4(1\text{M}) = 0.98$. These results show that, while the size of the dataset has an impact on unicity, the decrease in unicity is slow.

To further study how unicity decreases with dataset size and whether it decreases sufficiently in population-scale datasets, we propose a simple statistical model taking into account three population-wide marginal distributions—circadian (P_C), frequency (P_F), and activity (P_A)—along with the network of mobile phone antennas in a country. Using solely these quantities, the model is able to replicate the observed decrease in unicity with dataset size.

Figure 2 displays the information extracted from the dataset, three distributions, and the antenna network. (P_C) characterizes the circadian cycle, the overall likelihood of a record to occur at a given time in a week. The existence of circadian cycles is well documented in the computational social science literature,[22,23,25,26] and we use their empirical form in the model. The frequency distribution, (P_F), is the relative overall likelihood of a location to be visited. This distribution too has been studied before and has been widely shown to be well approximated by a power-law distribution,[44–48] as is also the case here (Figure 2B, $R^2 = 0.99$). The activity distribution, (P_A), captures the number

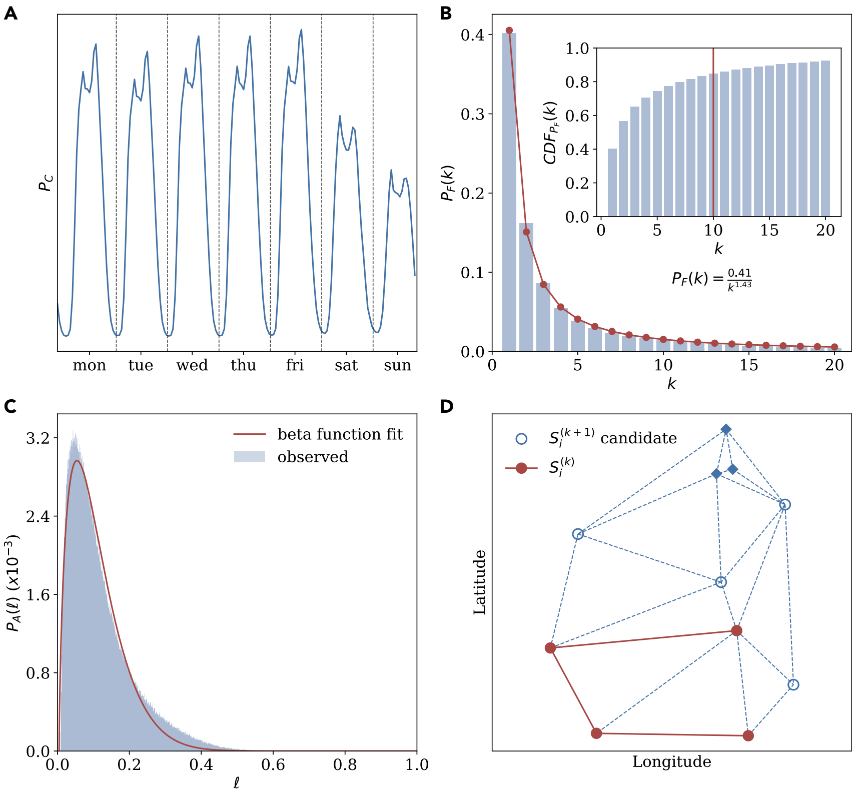

Figure 2. Inputs to the unicity model

(A) The circadian distribution, P_C.

(B) The frequency distribution, P_F, along with a power law fit (solid line, $R^2 = 0.99$). The inset displays the cumulative distribution with 85% of activity captured by the top 10 locations.

(C) The activity distribution, P_A, indicating the distribution of the number of records per trajectory along with a β distribution fit (solid line, $R^2 = 0.98$).

(D) Illustration of the sub-graph sampling method used to generate an antenna set S_i where $S_i^{(k)} \in S_i$. The underlying antenna network is represented by dotted lines. The filled nodes (circles) correspond to locations already selected, while the hollow nodes are potential locations that could be selected next ($S_i^{(k+1)}$ candidates) (see Supplemental information for detailed algorithm). Remaining locations are represented by filled diamonds.

of records $\ell_{(i)} = |D_i|$ that appear in each user trace. We approximate it here with a β distribution ($\alpha = 1.72$, $\beta = 14.7$, $R^2 = 0.98$). Finally, S_i is the set of locations visited by person i. It is a sub-graph sampled from the Delaunay tessellation of the antenna coordinates ($\mathcal{L}$) in the dataset (see Supplemental information for the detailed algorithm).

In short, for each user, our model samples a list of 10 connected antennas $(S_1, \ldots, S_{10})$ on the network and an activity (number of records in the user's trace), $A \sim P_A$. Each record's timestamp C and position X is then sampled according to the circadian distribution $C \sim P_C$ and $X = S_K$, $K \sim P_F$. This model is formally defined in the Experimental procedures.

Figure 1A shows that our simple statistical model closely follows the empirical measure of unicity from 1 to 1M people (dashed and solid lines). Using the model, we then study how unicity is likely to evolve as the size of the dataset increases to 20M people (Figure 1B). For $N = 20$M, our model estimates unicity with three points to be close to $\widehat{\epsilon}_3(20\text{M}) = 0.93$, while knowing one more point would increase this to the region of $\widehat{\epsilon}_4(20\text{M}) = 0.99$. This is a stark difference with the linear extrapolation made by El Emam,[42] who reports a unicity of 0.01 with four points (we replicate El Emam's method in the discussion and display our results for up to 60M people in the Supplemental information).

The model provides good evidence that unicity is likely to remain high even in datasets as large as 20M people. For further evidence, we prove that the decrease in unicity with increasing dataset size follows a convex form, and use this result to provide a lower bound on unicity in large datasets. We show in the Supplemental information that the unicity of a dataset of size N can be expressed as a sum of convex functions of N, and is thus convex.

This builds on two assumptions: (1) there exists an underlying trajectory distribution T_X from which all trajectories $D_i \in D$ are sampled and (2) all trajectories are independent of one another, $D_i \perp\!\!\!\perp D_j$. The first assumption states that an underlying distribution for trajectories exists. Such a distribution would also capture correlations between individuals on a large scale (e.g., commuting patterns, cities, weekends). The second assumption presumes that the correlation between specific individuals is negligible when estimating unicity of large datasets.

A direct consequence of unicity being a strictly decreasing convex function is that it will be lower bounded by its linear tangent (treating unicity as a function of a real-valued N):

$$\epsilon(D(N)) \geq \epsilon(D(N')) + (N - N') \cdot \left.\frac{d\epsilon}{dN}\right|_{N=N'}. \quad \text{(Equation 1)}$$

Re-arranged and expressed for discrete values, this gives a lower bound for unicity:

$$\epsilon(D(N')) - \epsilon(D(N)) \leq (N - N') \cdot (\epsilon(D(N' - 1)) - \epsilon(D(N'))). \quad \text{(Equation 2)}$$

Using the tangent to the empirical unicity curves estimated by discrete difference over the range of $N \in [0.9\text{M}, 1\text{M}]$, we obtain a lower bound of 0.73 for $\epsilon_4(20\text{M})$ and 0.9 for $\epsilon_5(20\text{M})$ (Figure 1B, dotted lines).

Our results show that unicity decreases slowly with the size of the dataset and that it, very likely, remains high even in population-scale datasets. This refutes previous claims that privacy is preserved in population-scale datasets, instead showing the risk of re-identification to be high. Modern location datasets have a great potential to improve our society, for example, by training AI algorithms, but robust privacy engineering solutions are needed to use them safely.

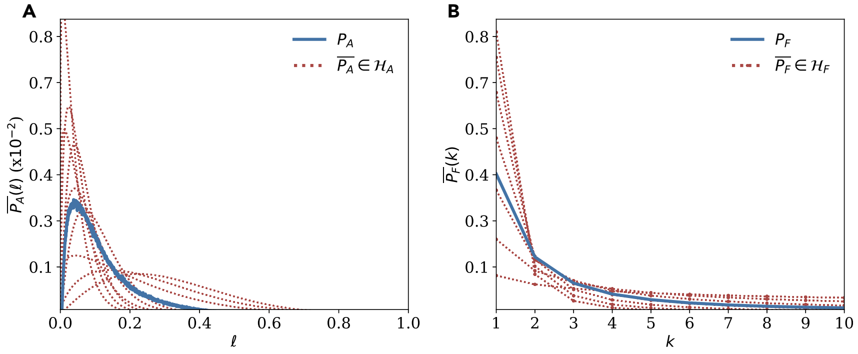

Figure 3. Range of distributions studied for the sensitivity analysis

The ranges of perturbed activity $\overline{P_A}$ (A) and frequency $\overline{P_F}$ (B) distributions are displayed (dotted lines) along with their empirical forms (solid lines).

DISCUSSION

Taken together, these results show that the scale of a dataset does not prevent re-identification. Human mobility, much like a physical fingerprint, is highly unique and can be used to find a person across mobility datasets.

Legally, the European Union (EU) General Data Protection Regulation sets a high threshold for what constitutes anonymous data, namely that the individual should not be identifiable taking into account both the "available technology at the time of the processing" but also future "technological developments" (Recital 26). The Article 29 Working Party, the predecessor to the European Data Protection Board, in its guidance sets out three criteria to assess whether a dataset is anonymous, singling out, linkability, and inference[49] with the former two being directly applicable here. As an example, the Centre for Humanitarian Data of the United Nations (UN OCHA) adopted 5% as a threshold for what constitutes an acceptable re-identification risk.[50] Even our lower bound of 22% far exceeds this liberal threshold.

Finally, here we study the unicity of location datasets with a spatial resolution of $\approx$1 km^2 and a temporal resolution of an hour. Fine-grained GPS data are likely to lead to even higher values of unicity, and previous research has shown that, in general, de-identification methods do not meaningfully reduce the risk of re-identification. For instance, research[32,34] has shown that reducing the spatial and temporal resolution of the data further only slowly decreases the risk, while another study[51] concluded that location data "show poor anonymizability [as measured by k-anonymity], i.e., require important spatial and temporal generalization in order to slightly improve user privacy".

Ensuring that these data can be accessed and used broadly is of paramount importance, but this should not come at the expense of people's privacy. A range of privacy engineering techniques allowing data to be used while giving individuals strong privacy guarantees have been developed and are starting to be used.[52–54] As standards for anonymization are being redefined, in the EU and around the world, it is essential for them to emphasize the strong limits of de-identification, possibly banning the uncontrolled release of individual-level de-identified data, and to give guidance on the use of modern privacy-engineering solutions.

In the next three sections we discuss the underlying assumptions of the unicity model and some considerations regarding the sensitivity of our results and, finally, include a discussion on previous estimates of unicity.

Assumptions underpinning the simple unicity model

We here evaluate the four assumptions underpinning the simple unicity model we present.

First, the model treats each of the four inputs in Figure 2 as independent of one another. Considering them, or some of them, jointly might further improve the model. This would, however, also increase its complexity and, therefore, its sensitivity to small changes in the data. Although further exploration would be interesting, we consider that the simple model approximates the decrease in unicity with increasing dataset size well enough to support our conclusion that unicity is unlikely to be low even in population-scale datasets.

Second, our model uses input distributions extracted from a dataset of 1M people to study the unicity of datasets with up to 60M people (see Supplemental information). This assumes that these distributions estimated from a smaller sample are representative of the larger sample (i.e., the estimation of the distributions has converged). We show that this is a reasonable assumption by instantiating our model $\mathcal{M}$ with distributions extracted from samples of sizes significantly smaller than 1M, and showing that the unicity results remain largely unchanged (Figure S5 in Supplemental information). We also perform a sensitivity analysis to evaluate the impact of broad variations on these input distribution on our results (see next subsection).

Third, the model assumes each trajectory to contain at most one unique location. This allows for the mean frequency distribution (P_F) to be used in the modeling process (Figure 2B). As seen in the inset of Figure 2B, more than 85% of the activity in the average trajectory is captured by the top 10 locations visited. Furthermore, we find that P_F changes only slightly when the number of unique locations is altered, and that our conclusions are not influenced by this choice.

Finally, our model assumes that the set of locations appearing in each trajectory can be described by a connected planar subgraph of the underlying antenna network. We believe this to be a reasonable assumption, as previous work suggests that subgraphs spanned by each trajectory in human mobility are highly localized, with the distribution $P(r_g)$ of the radius of gyration—a metric for how far people tend to travel on average—following a power law with increasing radius.[55]

Sensitivity analysis

Our simple statistical model for unicity takes as input three distributions. However, these distributions may vary depending on specifics of the dataset, such as the country where it was collected or the sources of location information. Here we perform a sensitivity analysis to ensure the robustness of our model to even broad changes to the distributions.

We first perturb the P_A and P_F distributions (Figure 3) around their empirical forms using a scaled earth mover's distance as

Table 1. Summary of unicity results at $N = 20M$ as per the sensitivity analysis

	ϵ_2	ϵ_3	ϵ_4	ϵ_5
Mean	0.307	0.735	0.876	0.935
Standard deviation	0.175	0.216	0.159	0.113
Minimum	0.071	0.260	0.431	0.544
Maximum	0.704	0.997	1	1

the guiding metric (see Supplemental information for details). The P_C distribution, on the other hand, has been shown to be very stable across datasets[22–26] and we thus keep it constant throughout our analysis.

These distributions are combined to produce 63 different instantiations of the unicity model (Figure S2). Table 1 summarizes the unicity values for models using the broad range of distributions in Figure 3, at a dataset size of 20M trajectories (see Supplemental information for 60M results). Note that the lowest unicity values across all instantiations of the model are still high, with $Min(\epsilon_4(20M)) = 43.1\%$ and would still be considered as putting people's privacy at risk.

Further, we study how certain aspects of human mobility contribute to unicity. Starting from empirical user location traces $D_i = (X_i, C_i)$, first, we find that removing the association between times (C_i) and locations (X_i), by shuffling the vectors and recombining them, only slightly affects unicity values (Figure S4A). Specifically, consider a dataset D' composed of trajectories $D\prime_i = (X\prime_i, C\prime_i)$ such that:

$$X\prime_i = \sigma_i(X_i),$$

$$C\prime_i = \pi_i(C_i),$$

where σ_i and π_i refer to random permutations of the spatial and temporal components of D_i. This only marginally affects unicity, showing that unicity does not depend on the specific places being visited at specific times, as long as those times and places appear in the trace with their respective frequencies independently.

Second, we replace the set of locations in each trajectory with uniformly picked locations. Instead of using the sub-graph sampling method displayed in Figure 2D, we populate each S_i with antennas picked from the entire set of locations $\mathcal{L}$ uniformly at random. We find that this leads to unicity being overestimated (Figure S4D).

Third, replacing P_C or P_F with uniform distributions (Figures S4B and S4C) or attempts to model unicity using a simple combinatorial model (Figure S3) also cause the model to overestimate unicity. These demonstrate the importance of all three distributions and the underlying geography to correctly capture the unicity of mobility datasets.

This analysis, combined with the relative simplicity and generality of the unicity model, strongly suggest that our results would generalize to any location dataset. Likewise, the strong underlying combinatorial effect that underpins unicity combined with previous research[34–36] suggests that unicity will similarly decrease slowly in other types of high-dimensional data.

El Emam's method

El Emam[42] proposed a method (hereafter the EE method) to estimate the uniqueness of a population-size (N) dataset given the unicity $\epsilon(m)$ of a smaller sample dataset of size m. Using this method, he estimates that the uniqueness for a population of size $N = 22 \cdot 10^6$ is about 1%, given a uniqueness of 90% of a sample of size $m = 22 \cdot 10^6$ of the same dataset. This estimate forms the basis for his claim that uniqueness is low in large-scale datasets.

We here show that the EE method (1) is unrealistic and (2) provably gives the lowest possible estimate for the risk in the larger dataset, and that (3) by using our dataset, we observe that the real empirical unicity is significantly higher than the upper bound given by the EE method.

First, the method is unrealistic, as it effectively generates a dataset D of size N where a fraction α of records are unique, while all the other records are identical to exactly one and only one other record. The parameter α is selected such that the expected estimated uniqueness on a sample of size m, which we denote by $\nu_D(m)$, is equal to the empirical unicity. This assumes that users in the real mobility dataset are either unique or exact duplicates of another user.

Second, we prove in the Supplemental information that the risk estimated by the EE method will be lower or equal to the risk of *any* other dataset of size N, as this estimate is an *affine* function of m. In other terms, this method will *always* return the absolute lowest possible estimate of the risk.

Third, we apply the EE method to our dataset and show that its estimate of the risk is significantly lower than the real empirical value, leading to the risk of re-identification being strongly underestimated. For a dataset of 200,000 people, we empirically observe an $\epsilon_2(200K) = 0.86$. Using this number, El Emam's method would estimate the risk of a larger 1M person dataset to be $\epsilon_2(1M) = 0.3$, while the correct empirical value is ≈ 0.7.

Taken together, our results cast serious doubt on the validity of the EE method to carry out risk assessments.

EXPERIMENTAL PROCEDURES

Resource availability

Lead contact

Further information and requests for resources should be directed to and will be fulfilled by the lead contact, Yves-Alexandre de Montjoye (demontjoye@imperial.ac.uk).

Materials availability

There are no physical materials associated with this study.

Data and code availability

Due to reasons of confidentiality and user privacy, we cannot share the raw data. However, we can make available all the input distributions and raw empirical results upon request for purposes of reproducibility.

The code used for all experiments is available at: github.com/computationalprivacy/scaling-unicity.

The unicity model in detail

We propose a simple statistical model $\mathcal{M}$ taking into account three population-wide distributions: activity (P_A), circadian (P_C), and frequency (P_F). This model samples location traces for each user independent of other users to estimate unicity of a dataset of size N. These location traces are then grouped together to compute unicity.

Formally, the model $\mathcal{M}$ can be written as:

$$\mathcal{M}(P_A, P_C, P_F, \mathcal{L}, N) = D = (D_1, \ldots, D_N). \quad \text{(Equation 3)}$$

Each $D_i \in D$ is a location trace for a unique user, represented as a list of L_i records $(X_i^{(j)}, C_i^{(j)})_{j=1}^{L_i}$. The length L_i of trace D_i is sampled from the empirical activity distribution P_A:

$$\mathbb{P}[L_i = \ell] = P_A(\ell). \quad \text{(Equation 4)}$$

The timestamps of each record in a trace, $(C_i^{(j)})_{j=1}^{L_i}$, are sampled independent of the empirical circadian distribution P_C:

$$\mathbb{P}\left[C_i^{(j)} = c\right] = P_C(c) \quad \forall j \in \{1, \ldots, L_i\}. \quad \text{(Equation 5)}$$

For the spatial component, for each user, a connected sub-graph S_i of size 10 is first sampled from the Delaunay tessellation of the antenna coordinates $\mathcal{L}$. This sub-graph is then randomly ordered as a list, which we denote by $S_i = (S_i^{(k)})_{k=1}^{10}$ with a slight abuse of notations. Finally, the locations of the records $X_i^{(j)} \in X_i$ are sampled independent of S_i according to the empirical frequency distribution P_F:

$$\mathbb{P}\left[X_i^{(j)} = S_i^{(k)}\right] = P_F(k) \quad \forall j \in \{1, \ldots, L_i\}. \quad \text{(Equation 6)}$$

Note that when the size of the dataset N sampled by our model $\mathcal{M}$ increases, this corresponds to sampling more individuals from the same underlying geography. This is what we mean throughout this work when we increase the size of the dataset, e.g., in unicity curves (Figure 1): we consider the dataset to be a growing sample from the same underlying population.

SUPPLEMENTAL INFORMATION

Supplemental information can be found online at https://doi.org/10.1016/j.patter.2021.100204.

ACKNOWLEDGMENTS

The authors would like to thank Shubham Jain for their comments on the codebase, and Ana-Maria Cretu, Andrea Gadotti, Shubham Jain, Thibaut Lienart, Axel Oehmichen, Luc Rocher, and Arnaud Tournier for their invaluable comments on the manuscript. We acknowledge support from the Agence Française de Développement as part of its financial assistance to the OPAL project.

AUTHOR CONTRIBUTIONS

A.F. designed and performed the experiments, built the models, helped with the mathematical results, and drafted the manuscript. F.H. derived the mathematical results, advised on model construction, and revised the manuscript. Y-A.d.M. designed the experiments and revised the manuscript.

DECLARATION OF INTERESTS

The authors declare no competing financial interests.

Received: August 24, 2020
Revised: November 27, 2020
Accepted: January 7, 2021
Published: February 12, 2021

REFERENCES

1. Vodafone. (2018). Vodafone UK's company history and achievements. https://www.vodafone.co.uk/about-us/company-history/.
2. Lomas, N. (2017). How "anonymous" wifi data can still be a privacy risk (TechCrunch). http://tcrn.ch/2ywXGdy.
3. Deville, P., Linard, C., Martin, S., Gilbert, M., Stevens, F.R., Gaughan, A.E., Blondel, V.D., and Tatem, A.J. (2014). Dynamic population mapping using mobile phone data. Proc. Natl. Acad. Sci. U S A *111*, 15888–15893.
4. Ratti, C., Frenchman, D., Pulselli, R.M., and Williams, S. (2006). Mobile landscapes: using location data from cell phones for urban analysis. Environ. Plann. B Plann. Des. *33*, 727–748.
5. Wesolowski, A., Eagle, N., Tatem, A.J., Smith, D.L., Noor, A.M., Snow, R.W., and Buckee, C.O. (2012). Quantifying the impact of human mobility on malaria. Science *338*, 267–270.
6. Gomes, M.F., Pastore, Y., Piontti, A., Rossi, L., Chao, D., Longini, I., Halloran, M.E., and Vespignani, A. (2014). Assessing the International spreading risk associated with the 2014 west African Ebola outbreak. PLoS Currents *6*, ecurrents.outbreaks.cd818f63d40e24aef769d-da7df9e0da5.
7. Mari, L., Bertuzzo, E., Righetto, L., Casagrandi, R., Gatto, M., Rodriguez-Iturbe, I., and Rinaldo, A. (2012). Modelling cholera epidemics: the role of waterways, human mobility and sanitation. J. R. Soc. Interface *9*, 376–388.
8. Bajardi, P., Poletto, C., Ramasco, J.J., Tizzoni, M., Colizza, V., and Vespignani, A. (2011). Human mobility networks, travel restrictions, and the global spread of 2009 H1n1 pandemic. PLoS One *6*, e16591.
9. Merler, S., and Ajelli, M. (2009). The role of population heterogeneity and human mobility in the spread of pandemic influenza. Proc. Biol. Sci. *277*, 557–565.
10. Aktay, A., Bavadekar, S., Cossoul, G., Davis, J., Desfontaines, D., Fabrikant, A., Gabrilovich, E., Gadepalli, K., Gipson, B., Guevara, M., et al. (2020). Google COVID-19 community mobility reports: anonymization process description (version 1.0). arXiv, preprint arXiv:2004.04145.
11. Steele, J.E., Sundsøy, P.R., Pezzulo, C., Alegana, V.A., Bird, T.J., Blumenstock, J., Bjelland, J., Engø-Monsen, K., de Montjoye, Y.A., Iqbal, A.M., et al. (2017). Mapping poverty using mobile phone and satellite data. J. R. Soc. Interface *14*, 20160690.
12. Toole, J.L., Lin, Y.-R., Muehlegger, E., Shoag, D., González, M.C., and Lazer, D. (2015). Tracking employment shocks using mobile phone data. J. R. Soc. Interface *12*, 20150185.
13. Wesolowski, A., Eagle, N., Noor, A.M., Snow, R.W., and Buckee, C.O. (2013). The impact of biases in mobile phone ownership on estimates of human mobility. J. R. Soc. Interface *10*, 20120986.
14. Madden, M., Rainie, L., Zickuhr, K., Duggan, M., and Smith, A. (2014). Public Perceptions of Privacy and Security in the Post-Snowden Era, *12* (Pew Research Center).
15. Blumenstock, J., Cadamuro, G., and On, R. (2015). Predicting poverty and wealth from mobile phone metadata. Science *350*, 1073–1076.
16. Li G., Yu L., Ng W.S., Wu W., and Goh S.T. Predicting Home and Work Locations Using Public Transport Smart Card Data by Spectral Analysis. In 2015 IEEE 18th International Conference on Intelligent Transportation Systems, pages 2788–2793, Gran Canaria, Spain, September 2015. IEEE.
17. Ashbrook D. and Starner T. Learning significant locations and predicting user movement with GPS. In Proceedings. Sixth International Symposium on Wearable Computers, pages 101–108, Seattle, WA, USA, 2002. IEEE.
18. Isaacman, S., Becker, R., Cáceres, R., Kobourov, S., Martonosi, M., Rowland, J., and Varshavsky, A. (2011). Identifying important places in people's lives from cellular network data. In Pervasive Computing, Volume 6696 of Lecture Notes in Computer Science, K. Lyons, J. Hightower, and E.M. Huang, eds. (Springer Berlin Heidelberg), pp. 133–151.
19. Mahmud, J., Nichols, J., and Drews, C. (2014). Home location identification of Twitter users. ACM Trans. Intell. Syst. Technol. *5*, 47.
20. Li R., Wang S., Deng H., Wang R, and Chen-Chuan Chang K. Towards Social User Profiling: Unified and Discriminative Influence Model for Inferring Home Locations. In Proceedings of the 18th ACM SIGKDD International Conference on Knowledge Discovery and Data Mining, pages 1023–1031, New York, NY, USA, 2012. ACM.
21. Cho E., Myers S.A., and Leskovec J. Friendship and Mobility: User Movement in Location-based Social Networks. In Proceedings of the 17th ACM SIGKDD International Conference on Knowledge Discovery and Data Mining, pages 1082–1090, New York, NY, USA, 2011. ACM.
22. Monsivais, D., Ghosh, A., Bhattacharya, K., Dunbar, R.I.M., and Kaski, K. (2017). Tracking urban human activity from mobile phone calling patterns. PLoS Comput. Biol. *13*, e1005824.

CellPress
OPEN ACCESS

23. Monsivais, D., Bhattacharya, K., Ghosh, A., Dunbar, R.I.M., and Kaski, K. (2017). Seasonal and geographical impact on human resting periods. Sci. Rep. *7*, 10717.

24. Kondor, D., Hashemian, B., de Montjoye, Y.-A., and Ratti, C. (2020). Towards matching user mobility traces in large-scale datasets. In IEEE Transactions on Big Data, *6*, p. 1, 714-726.

25. Hasan S., Zhan X., and Ukkusuri S.V. Understanding Urban Human Activity and Mobility Patterns Using Large-scale Location-based Data from Online Social Media. In Proceedings of the 2Nd ACM SIGKDD International Workshop on Urban Computing, UrbComp '13, pages 6:1–6:8, New York, NY, USA, 2013. ACM.

26. Ahas, R., Aasa, A., Silm, S., and Tiru, M. (2010). Daily rhythms of suburban commuters' movements in the Tallinn metropolitan area: case study with mobile positioning data. Transport. Res. C Emerg. Tech. *18*, 45–54.

27. Felbo, B., Sundsøy, P., Pentland, A., Lehmann, S., and de Montjoye, Y.-A. (2017). Modeling the temporal nature of human behavior for demographics prediction. In Machine learning and knowledge discovery in databases, volume 10536 of lecture notes in computer science (Springer), pp. 140–152.

28. de Montjoye, Y.-A., Quoidbach, J., Robic, F., and Pentland, A.S. (2013). Predicting personality using novel mobile phone-based metrics. In International conference on social computing, behavioral-cultural modeling, and prediction (Springer), pp. 48–55.

29. Onnela, J.-P., Saramäki, J., Hyvönen, J., Szabó, G., Lazer, D., Kaski, K., Kertész, J., and Barabási, A.-L. (2007). Structure and tie strengths in mobile communication networks. Proc. Natl. Acad. Sci. U S A *104*, 7332–7336.

30. Onnela, J.-P., Saramäki, J., Hyvönen, J., Szabó, G., De Menezes, M.A., Kaski, K., Barabási, A.-L., and Kertész, J. (2007). Analysis of a large-scale weighted network of one-to-one human communication. New J. Phys. *9*, 179.

31. Krumme, C., Llorente, A., Cebrian, M., Pentland, A.S., and Moro, E. (2013). The predictability of consumer visitation patterns. Sci. Rep. *3*, 1645.

32. de Montjoye, Y.-A., Hidalgo, C.A., Verleysen, M., and Blondel, V.D. (2013). Unique in the crowd: the privacy bounds of human mobility. Sci. Rep. *3*, 1376.

33. Pellungrini, R., Pappalardo, L., Pratesi, F., and Monreale, A. (2017). A data mining approach to assess privacy risk in human mobility data. ACM Trans. Intell. Syst. Technol. *9*, 31.

34. Achara, J.P., Acs, G., and Castelluccia, C. (2015). On the Unicity of Smartphone Applications (ACM Press), pp. 27–36.

35. Sekara, V., Mones, E., and Jonsson, H. (2018). Temporal limits of privacy in human behavior. arXiv, preprint arXiv:1806.03615.

36. de Montjoye, Y.-A., Radaelli, L., Singh, V.K., and Pentland, A.P. (2015). Unique in the shopping mall: on the reidentifiability of credit card metadata. Science *347*, 536–539.

37. Xu, Y., Belyi, A., Bojic, I., and Ratti, C. (2018). Human mobility and socioeconomic status: analysis of Singapore and Boston. Comput. Environ. Urban Syst. *72*, 51–67.

38. Deußer C., Passmann S., and Strufe T. Browsing unicity: On the limits of anonymizing web tracking data. In 2020 IEEE Symposium on Security and Privacy (SP), pages 777–790. IEEE, 2020.

39. Narayanan, A., and Shmatikov, V. (2008). Robust De-anonymization of Large Sparse Datasets (IEEE), pp. 111–125.

40. Riederer, C., Kim, Y., Chaintreau, A., Korula, N., and Lattanzi, S. (2016). Linking users across domains with location data: Theory and validation. In Proceedings of the 25th International Conference on World Wide Web (International World Wide Web Conferences Steering Committee), pp. 707–719.

41. Snchez, D., Martnez, S., and Domingo-Ferrer, J. (2016). Comment on "Unique in the shopping mall: on the reidentifiability of credit card metadata". Science *351*, 1274.

42. El Emam, K. (2015). On Re-identification: Not Really Unique in the Shopping Mall.

43. Barth-Jones, D., El Emam, K., Bambauer, J., Cavoukian, A., and Malin, B. (2015). Assessing data intrusion threats. Science *348*, 194–195.

44. Pappalardo, L., and Simini, F. (2018). Data-driven generation of spatio-temporal routines in human mobility. Data Min. Knowl. Discov. *32*, 787–829.

45. Gonzlez, M.C., Hidalgo, C.A., and Barabási, A.-L. (2008). Understanding individual human mobility patterns. Nature *453*, 779.

46. Alessandretti, L., Sapiezynski, P., Sekara, V., Lehmann, S., and Baronchelli, A. (2018). Evidence for a conserved quantity in human mobility. Nat. Hum. Behav. *2*, 1.

47. Song, C., Koren, T., Wang, P., and Barabási, A.-L. (2010). Modelling the scaling properties of human mobility. Nat. Phys. *6*, 818–823.

48. Hasan, S., Schneider, C.M., Ukkusuri, S.V., and González, M.C. (April 2013). Spatiotemporal patterns of urban human mobility. J. Stat. Phys. *151*, 304–318.

49. Article 29 Data Protection Working Party (2014). Opinion 05/2014 on Anonymisation Techniques (European Commission).

50. Centre for Humanitarian Data of the United Nations Office for the Coordination of Humanitarian Affairs (2019). Guidance Note Series on Data Responsibility on Humanitarian Action. Note 1: Statistical Disclosure Control (United Nations).

51. Gramaglia, M., and Fiore, M. (2014). On the anonymizability of mobile traffic datasets. arXiv, preprint arXiv:1501.00100.

52. Oehmichen, A., Jain, S., Gadotti, A., and de Montjoye, Y.-A. (2019). Opal: high performance platform for large-scale privacy-preserving location data analytics. In 2019 IEEE International Conference on Big Data (Big Data) (IEEE), pp. 1332–1342.

53. Mir, D.J., Isaacman, S., Cáceres, R., Martonosi, M., and Wright, R.N. (2013). Dp-where: Differentially private modeling of human mobility. In 2013 IEEE international conference on big data (IEEE), pp. 580–588.

54. Francis, P., Probst Eide, S., and Munz, R. (2017). Diffix: high-utility database anonymization. In Annual Privacy Forum (Springer), pp. 141–158.

55. Gonzalez, M.C., Hidalgo, C.A., and Barabasi, A.-L. (2008). Understanding individual human mobility patterns. Nature *453*, 779.

Patterns, Volume *2*

Supplemental information

The risk of re-identification remains high even in country-scale location datasets

Ali Farzanehfar, Florimond Houssiau, and Yves-Alexandre de Montjoye

Supplemental Experimental Procedures for:
The risk of re-identification remains high even in country-scale location datasets

1 Estimated unicity of larger datasets

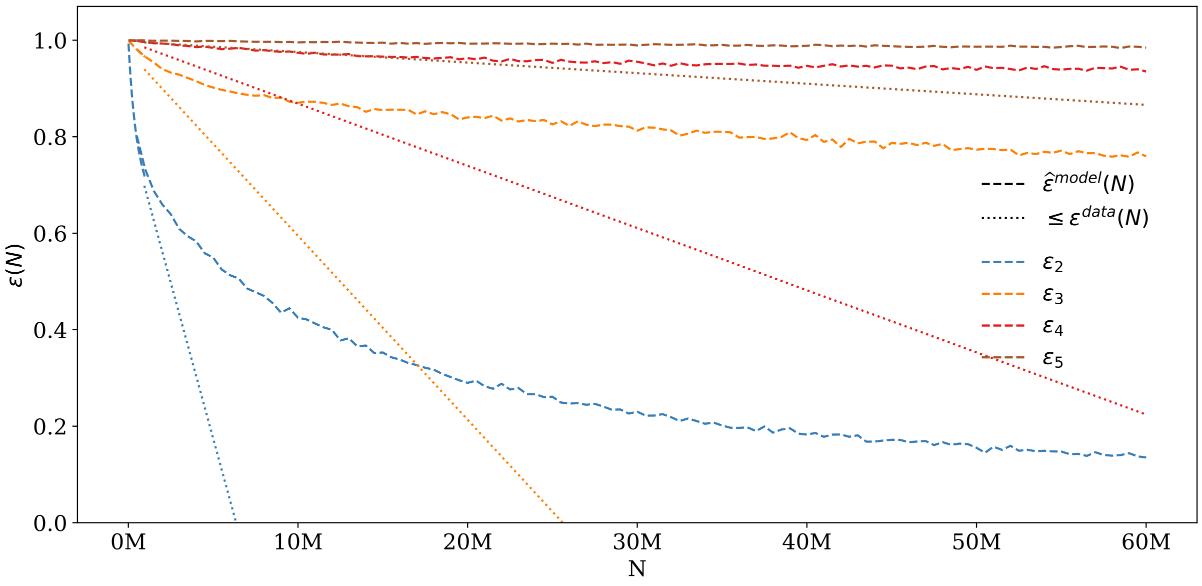

Fig S1 Estimated unicity values along a lower bound on unicity. The estimated unicity remains high even in large dataset. This is confirmed by the lower bound results.

Based on Figure S1, 93% of people would be uniquely identified in a dataset of 60M people using 4 points of auxiliary information with a lower bound at 22%. This bound increases to 87% when 5 points are available. These results re-enforce the conclusion of the main text that the risk of re-identification remains high even in country-scale location datasets.

2 Sensitivity analysis

The unicity model vary with both the frequency distribution (P_F), a power law, and the activity distribution (P_A), a Beta distribution. Broadly speaking, one can observe that:

- **Frequency distribution.** With increasing values of the exponent of the power law unicity decreases

- **Activity distribution.** Given a fitted Beta function $\beta(a, b)$:
  1. Unicity decreases with increasing values of a
  2. Unicity increases with increasing values of b

We here provide more details on the sensitivity analysis conducted in this work. In Figure 2 in the main text we saw that the empirical frequency and activity distributions are each approximated by a power-law and a beta function respectively. These are:

$$\overline{P_F}(k) = \frac{c}{k^\gamma} \tag{S1}$$

$$\overline{P_A}(l) = c \cdot l^{a-1} \cdot (1-l)^{b-1} \tag{S2}$$

Here, we perturb these distributions around their empirical fit. For each of the two, we change their fit parameters to obtain a set of new distributions $P_{\mathrm{q}} \in \mathcal{H}_q^{all}$ where $q \in \{A, F\}$ corresponds to either the frequency, F, or activity, A, distributions. $\mathcal{H}_q^{all}$ corresponds to the set of all possible distributions. The P_q included in this sensitivity analysis are picked such that the earth movers distance (also called first Wasserstein distance) (d_{emd}) between P_q and the observed distribution P_{obs}, is no more than 70% of its maximum distance given. While in general the earth movers distance between two probability distributions is not bounded, in our case, the distributions are defined over a discrete finite space and the distance is thus bounded by a finite number.

The set of distributions included in this analysis are a subset of the elements of $\mathcal{H}_q$ which is constructed as a random sample of $\mathcal{H}_q^{all}$. Therefore, in this analysis, we choose those $P_q \in \mathcal{H}_q \subset \mathcal{H}_q^{all}$ such that:

$$\mathcal{H}_q^{selected} = \left\{ P_q \in \mathcal{H}_q \mid d_{emd}(P_q, P_{obs}) \leq 0.7 \cdot \sup_{P_q \in \mathcal{H}_q} \{ d_{emd}(P_q, P_{obs}) \} \right\} \tag{S3}$$

Our estimate of the parameter values that satisfy this heuristic are included in table S1 and table S2. The constant values c are normalising factors and are only included here for the purposes of reproducibility. The resulting distributions are visible in Figure 3 in the main text.

Each combination of $\overline{P_A}$ and $\overline{P_F}$ yields a new instantiation of the model, generating 4 new sets of unicity estimates — unicity computed with 2, 3, 4 and, 5 points of auxiliary information. The plots in Figure S2 demonstrate that the reduction of unicity in each of these is very similar in form across all the different instantiations. The summary of these results is visible in table S3.

Table S1 $\overline{P_F}$ parameter values

γ	c
0.390	0.173
0.802	0.281
1.214	0.409
1.626	0.540
2.038	0.655
2.450	0.747
2.862	0.816

Table S2 $\overline{P_A}$ parameter values

a	b	c
1.186	5.142	0.0036
1.186	17.094	0.0146
1.186	29.046	0.0275
1.689	5.142	0.009
1.689	17.094	0.0639
1.689	29.046	0.1546
2.193	5.142	0.01927
2.193	17.094	0.2298
2.193	29.046	0.7137

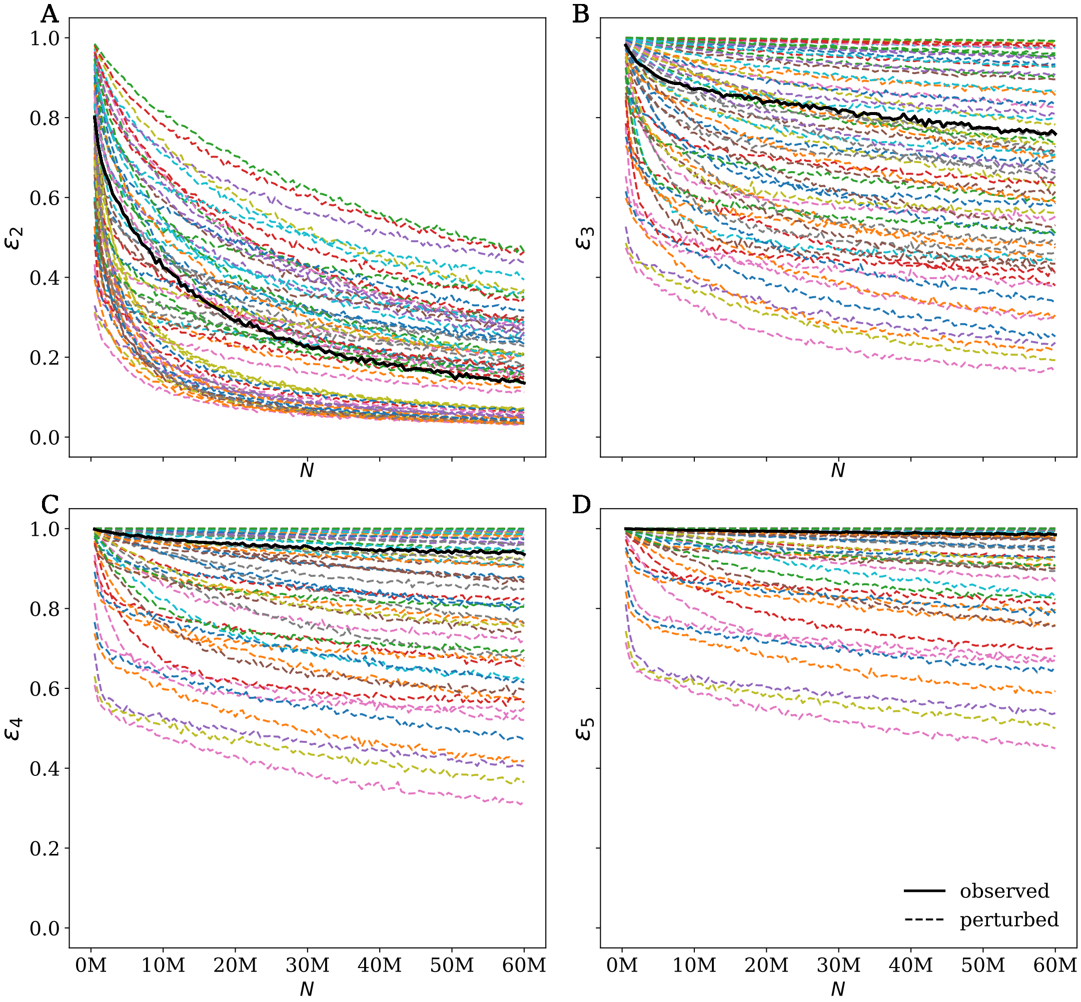

Fig S2 Unicity of trajectories generated using the 63 different perturbed input distributions (dashed) for ε_2 (**A**), ε_3 (**B**), ε_4 (**C**) and, ε_5 (**D**). The solid black line is the unicity model calibrated with the non-perturbed observed input distributions.

	ε_2	ε_3	ε_4	ε_5
Mean	0.182	0.656	0.83	0.908
Standard deviation	0.121	0.24	0.192	0.129
Minimum	0.033	0.162	0.317	0.449
Maximum	0.464	0.991	1	1

Table S3 Summary of unicity results at $N = 60M$ as per the sensitivity analysis.

3 Data set

Our dataset consists of the location traces of close to 1M individuals who have visited at least 10 distinct locations over 3 months for one telecommunication operator. Each record in the data consists of a timestamp with hourly granularity, the GPS coordinates of the antenna the user was connected to the most during the hour, and a unique pseudonym. The records are generated when an individual interacts with the mobile phone network (i.e. makes/receives a call or sends/receives a text). Further, the data contains nearly 6000 antennas that span the whole of the country. Antennas in our database are distributed throughout the country that has quite a heterogeneous geography. The median area covered by an antenna is 16 km^2 with smaller antennas covering areas closer to 0.05 km^2. More than 50% of antennas cover areas that are less than 10 km^2 and the distribution of subscribers across the country roughly follows its population density. Finally, for each trajectory in the data, multiple antennas can be associated to one hour (e.g. multiple calls made within the same hour). In such a case, we pick the most popular antenna within the hour with ties broken randomly.

4 Estimating unicity

Computing the exact unicity of a data set D is extremely computationally expensive (see equation S5). In line with previous work, we estimate unicity by randomly sampling a smaller data set of users $Q \subset D$ and computing the fraction of trajectories in Q that are unique in D given p randomly selected points from each user in Q. Namely, from each trajectory $T^{(i)} \in Q$, p points are picked uniformly at random, labelled $A_p^{(i)} \subset T^{(i)}$. The unicity estimate ε_p is then the fraction of trajectories in Q that are unique in the entire data set D, given $A_p^{(i)}$ as auxiliary information (see algorithm S2 of reference [36] of the main text).

5 Algorithm for graph sub-sampling

One of the four components of the unicity model is a set of 10 distinct antennas S_i visited by the trajectory i (Figure 2D). This location set is obtained following the procedure outlined below:

1. Use the geographic coordinates of all antennas in the location set $\mathcal{L}$ to generate the Delaunay tessellation $\mathcal{G}$ (Full diamonds in Figure 2D of the main text).

2. Pick a starting antenna $S_i^{(j)}$ uniformly at random from $\mathcal{L}$.

3. Add $S_i^{(j)}$ to the set $S_i = \{S_i^{(j)}\}$ (Full red circles in Figure 2D of the main text).

4. Find all the neighbouring antennas to $S_i^{(j)}$ according to $\mathcal{G}$, denoted by $Neighbours(G, j)$.

5. Add all the locations in $Neighbours(G, j)$ to a choice set U_i (Hollow circles in Figure 2D of the main text).

6. Pick, uniformly at random, an antenna $S_i^{(k)}$ from $W_i = U_i \setminus S_i$, and add it to S_i.

7. Repeat steps 3 - 6 until $|S_i| = 10$.

This process effectively samples a connected sub-graph denoted by nodes S_i from the Delaunay tessellation G. This sub-graph will then be used as the set of locations visited by the user i in the unicity model $\mathcal{M}$.

Note that the above algorithm describes the procedure through which the location set S_i is produced. It does not imply that this is how users actually move from node to node.

6 Convexity of unicity

Our linear lower bound on unicity relies on the relationship between unicity and the size of the dataset to be convex. Here, we prove that as long as trajectories are independent and identically distributed, the expected unicity of a population, as a function of population size, follows such a form.

Let $\mathcal{T}$ the set of all possible trajectories. We assume $\mathcal{T}$ to be finite, an assumption which agrees with our empirical setting as the number of possible points (number of hour-location combinations) that could constitute a trajectory is finite, as is the set of all points. We define a data set D as a collection of N trajectories: $D = (D_1, \ldots, D_N),\ D_i \subseteq \mathcal{T}$. A unicity model $\mathcal{M}$ is a randomised mechanism that for a value of N generates a data set D of size N given that:

1. All trajectories $D_i \in D$ are sampled from the *same* distribution T_X

2. All trajectories are sampled *independently* from T_X, meaning $D_i \perp\!\!\!\perp D_j$

The first assumption states that an underlying distribution for population trajectories T_X exists, and it captures correlation between individuals on a large-scale (e.g. commuting patterns, cities,

weekends). The second assumption assumes that the correlation between specific individuals is negligible when estimating unicity.

We can thus write that a data set is a random variable $D = (D_1, \ldots, D_N)$, with $D_i \sim T_X$ and $D_i \perp\!\!\!\perp D_j$ if $i \neq j$. In the following notation, all random variables are represented by upper-case letters[1] and their realisations or possible values by lower-case letters[2].

Unicity is computed with knowledge of a random set of points in a trajectory. Given a set $A_p^{d_i} \subseteq d_i$ of p points *picked uniformly at random* from a trajectory d_i, we denote the probability that this trajectory $d_i \in d$ is unique in d as:

$$\mathbb{P}_{A_p^{d_i}}\left[A_p^{d_i} \not\subseteq d_j : \forall j \neq i\right] \tag{S4}$$

Unicity $\varepsilon_p(d)$ for a data set d is then defined as this probability (equation S4) averaged over all trajectories in a particular data set d. Let $N = |d|$, we thus have:

$$\varepsilon_p(d) = \frac{1}{N} \sum_{i=1}^{N} \mathbb{P}_{A_p^{d_i}}\left[A_p^{d_i} \not\subseteq d_j : \forall j \neq i\right] \tag{S5}$$

We are interested in the *expected unicity* of a data set D generated by our randomised mechanism $\mathcal{M}$. This is the expectation of equation S5 taken over all possible data sets d.

$$\bar{\varepsilon}_p(N) = \mathbb{E}_D\left[\varepsilon_p(D)\right] \tag{S6}$$

From here we can use equations S4 and S5 to obtain:

[1] $D_i, X \ldots$
[2] $d_i, x \ldots$

$$\bar{\varepsilon}_p(N) = \mathbb{E}_D\left[\varepsilon_p(D)\right] \tag{S7}$$

$$= \mathbb{E}_D\left[\frac{1}{N}\sum_{i=1}^{N}\mathbb{P}_{A_p^{D_i}}\left[A_p^{D_i} \not\subseteq D_j : \forall j \neq i\right]\right] \tag{S8}$$

$$= \frac{1}{N}\sum_{i=1}^{N}\mathbb{E}_D\left[\mathbb{P}_{A_p^{D_i}}\left[A_p^{D_i} \not\subseteq D_j : \forall j \neq i\right]\right] \tag{S9}$$

where in equation S9 we have substituted equation S4 while also specifying that the trajectories are now random variables as the expectation is taken over the data set D. Next we will expand this to obtain:

$$\bar{\varepsilon}_p(N) = \frac{1}{N}\sum_{i=1}^{N}\mathbb{E}_D\left[\mathbb{P}_{A_p^{D_i}}\left[A_p^{D_i} \not\subseteq D_j : \forall j \neq i\right]\right] \tag{S10}$$

$$= \frac{1}{N}\sum_{i=1}^{N}\sum_{d\in\mathcal{T}^N}\mathbb{P}[D=d]\cdot\mathbb{P}_{A_p^{d_i}}\left[A_p^{d_i} \not\subseteq d_j : \forall j \neq i\right] \tag{S11}$$

In equation S11 we used the definition of the expected value to obtain the sum over all possible data sets d. Let us expand this term (representing the other factor by a $\circ$):

$$\sum_{d\in\mathcal{T}^N}\mathbb{P}[D=d]\circ = \sum_{d\in\mathcal{T}^N}\mathbb{P}[D=(d_1,d_2,\ldots,d_N)]\circ \tag{S12}$$

$$= \sum_{d\in\mathcal{T}^N}\prod_{k=1}^{N}T_X(d_k)\circ \tag{S13}$$

$$= \sum_{d_1\in\mathcal{T}}\cdots\sum_{d_N\in\mathcal{T}}\prod_{k=1}^{N}T_X(d_k)\circ \tag{S14}$$

$$= \sum_{d_i\in\mathcal{T}}T_X(d_i)\sum_{d_1\in\mathcal{T}}\cdots\sum_{d_{i-1}\in\mathcal{T}}\sum_{d_{i+1}\in\mathcal{T}}\cdots\sum_{d_N\in\mathcal{T}}\prod_{k\neq i}T_X(d_k)\circ \tag{S15}$$

Going from S14 to S15, the ith component of the sum over the trajectories was pulled out to the front for later use. Going back to S9 we obtain:

$$\bar{\varepsilon}_p(N) = \frac{1}{N}\sum_{i=1}^{N}\sum_{d\in\mathcal{T}^N}\mathbb{P}[D=d]\,\mathbb{P}_{A_p^{d_i}}\left[A_p^{d_i}\not\subseteq d_j : \forall j\neq i\right] \tag{S16}$$

$$= \frac{1}{N}\sum_{i=1}^{N}\sum_{d_i\in\mathcal{T}} T_X(d_i)\sum_{d_1\in\mathcal{T}}\cdots\sum_{d_{i-1}\in\mathcal{T}}\sum_{d_{i+1}\in\mathcal{T}}\cdots\sum_{d_N\in\mathcal{T}}\prod_{k\neq i}T_X(d_k)\mathbb{P}_{A_p^{d_i}}\left[A_p^{d_i}\not\subseteq d_j : \forall j\neq i\right] \tag{S17}$$

At this stage equation S17 contains the expression for the expected value over $D\setminus\{D_i\}$ of the probability. We consolidate all sums to reflect this fact:

$$\bar{\varepsilon}_p(N) = \frac{1}{N}\sum_{i=1}^{N}\sum_{d_i\in\mathcal{T}} T_X(d_i)\sum_{d_1\in\mathcal{T}}\cdots\sum_{d_{i-1}\in\mathcal{T}}\sum_{d_{i+1}\in\mathcal{T}}\cdots\sum_{d_N\in\mathcal{T}}\prod_{k\neq i}T_X(d_k)\mathbb{P}_{A_p^{d_i}}\left[A_p^{d_i}\not\subseteq d_j : \forall j\neq i\right] \tag{S18}$$

$$= \frac{1}{N}\sum_{i=1}^{N}\sum_{d_i\in\mathcal{T}} T_X(d_i)\mathbb{E}_{D\setminus\{D_i\}}\left[\mathbb{P}_{A_p^{d_i}}\left[A_p^{d_i}\not\subseteq D_j : \forall j\neq i\right]\right] \tag{S19}$$

The probability expression essentially represents the chances that any randomly picked p points from d_i, appear in any other trajectory d_j. This can be computed as, by developing the probability over $A_p^{d_i}$:

$$\mathbb{P}_{A_p^{d_i}}\left[A_p^{d_i}\not\subseteq D_j : \forall j\neq i\right] = \sum_{a_p^{d_i}}\frac{1}{C_{|d_i|}^{p}}\mathbb{1}_{D_{j\neq i}}\left[a_p^{d_i}\not\subseteq D_j,\ \forall j\neq i\right] \tag{S20}$$

Where $C_{|d_i|}^{p}$ is the number of ways p points can be picked from the trajectory d_i. Inserting this back into equation S19 results in the following.

$$\bar{\varepsilon}_p(N) = \frac{1}{N}\sum_{i=1}^{N}\sum_{d_i\in\mathcal{T}} T_X(d_i)\mathbb{E}_{D\setminus\{D_i\}}\left[\sum_{a_p^{d_i}}\frac{1}{C_{|d_i|}^{p}}\mathbb{1}_{D_{j\neq i}}\left[a_p^{d_i}\not\subseteq D_j,\ \forall j\neq i\right]\right] \tag{S21}$$

Note that: 1) The expression above is agnostic to the index i which cancels with the leading factor of $\frac{1}{N}$ and, 2) The expected value of the indicator variable of a random event is the probability

of that event. Noting that $D \setminus \{D_i\}$ represents the same variable set as the ones impacting the indicator function $D_{j\neq i}$, we move in the expectation operator past the sum and the constant factor and therefore re-write equation S21 as:

$$\bar{\varepsilon}_p(N) = \sum_{d_i \in \mathcal{T}} T_X(d_i) \sum_{a_p^{d_i}} \frac{1}{C_{|d_i|}^p} \mathbb{P}_{D_{j\neq i}} \left[a_p^{d_i} \not\subseteq D_j \right] \tag{S22}$$

$$= \sum_{d_i \in \mathcal{T}} T_X(d_i) \sum_{a_p^{d_i}} \frac{1}{C_{|d_i|}^p} \prod_{j\neq i} \mathbb{P}_{D_j} \left[a_p^{d_i} \not\subseteq D_j \right] \tag{S23}$$

$$= \sum_{d_i \in \mathcal{T}} T_X(d_i) \sum_{a_p^{d_i}} \frac{1}{C_{|d_i|}^p} \prod_{j\neq i} \mathbb{P}_{X} \left[a_p^{d_i} \not\subseteq X \right] \tag{S24}$$

$$= \sum_{d_i \in \mathcal{T}} T_X(d_i) \sum_{a_p^{d_i}} \frac{1}{C_{|d_i|}^p} \left(\mathbb{P}_{X} \left[a_p^{d_i} \not\subseteq X \right] \right)^{N-1} \tag{S25}$$

In equation S23 we used the independence of different trajectories, and we used the fact that all trajectories are identically distributed in going from equation S24 to S25. Finally, with a change of dummy variables from d_i to y we obtain:

$$\bar{\varepsilon}_p(N) = \sum_{y \in \mathcal{T}} \frac{1}{C_{|y|}^p} T_X(y) \sum_{a_p^y} \left(\mathbb{P}_{X} \left[a_p^{y} \not\subseteq X \right] \right)^{N-1} \tag{S26}$$

Finally we note that the form of equation S26 is:

$$\bar{\varepsilon}_p(N) = \sum_{y \in \mathcal{T}} \frac{1}{C_{|y|}^p} T_X(y) \sum_{a_p^y} \left(\mathbb{P}_{X} \left[a_p^{y} \not\subseteq X \right] \right)^{N-1} \tag{S27}$$

$$= \sum_{y \in \mathcal{T}} \frac{1}{C_{|y|}^p} T_X(y) \sum_{x(y)} \left(f(x) \right)^{N-1} \tag{S28}$$

$$= \sum_{y \in \mathcal{T}} \frac{1}{C_{|y|}^p} T_X(y) g(y, N) \tag{S29}$$

We now show that $\bar{\varepsilon}_p(N)$ is a convex function of a real-valued N. From this, we obtain that the series $(\bar{\varepsilon}_p(N))_{N=1,2,\ldots}$ is a convex sequence (this is a direct consequence of the definition of convexity), which concludes the proof.

Functions of the form $f(m) = b^m$ are convex for all positive m and b. Further, the sum of convex functions is itself convex, and therefore $g(y, N)$ is convex in N in equation S29. Using this fact one more time we obtain that $\bar{\varepsilon}_p(N)$ must also be convex, since all elements being multiplied out in equations S27 to S29 are probabilities and are thus non-negative.

Finally, while our analysis considers real-valued functions, a direct consequence of this convexity result is that the sequence $\{\varepsilon(D(N))\}_{N=1,2,3...}$, which represents the population dependence of unicity for discrete population sizes, is also a convex sequence.

Given this result, the unicity of a dataset of size N will always obey the following relation (treating unicity as a function of a real-valued N):

$$\varepsilon(D(N)) \geq \varepsilon(D(N')) + (N - N') \cdot \left.\frac{d\varepsilon}{dN}\right|_{N=N'} \tag{S30}$$

The discrete equivalent of this expression replaces the differentiation operator by a difference operator. Re-arranged, this gives a lower bound for unicity:

$$\varepsilon\big(D(N')\big) - \varepsilon\big(D(N)\big) \leq (N - N') \cdot \Big(\varepsilon\big(D(N'-1)\big) - \varepsilon\big(D(N')\big)\Big) \tag{S31}$$

Unicity being a strictly decreasing convex function, it is lower-bounded by its linear tangent.

7 Estimating uniqueness by subsampling a larger dataset

El Emam [42] proposed a method to estimate the uniqueness of a population-size (N) dataset given its unicity $\varepsilon(m)$ for a smaller population size m: generating a dataset D of size N where a fraction α of records are unique, and all other records are identical to exactly one other record. The parameter α is chosen such that the expected estimated uniqueness on a sample of size m, which we denote by $\nu_D(m)$, is equal to the empirical unicity.

By applying this method to prior results [36, main text] ($m = 10^6$, $\varepsilon_4(m) = 0.9$), El Emam estimates that the real population uniqueness for a population of size $N = 22 \cdot 10^6$ is of about 1%. This is used as a basis to argue that the unicity of a sample strongly overestimates the uniqueness of population-scale data, a claim this paper refutes.

In the remainder of this section, we prove that El Emam's method computes a worst-case lower bound on the uniqueness of a population given a sample.

For this, we first show that for any dataset D of size N, the sample uniqueness as a function of the sample size m, $\nu_D(m)$, is a convex decreasing function of m. We then show that for the dataset created by El Emam's method, D_α, sample uniqueness $\nu_{D_2}(m)$ is an *affine* function of m. Hence, the uniqueness estimated with El Emam's method will be lower or equal to the uniqueness of *any* other dataset for all (with $m' > m$ including for $m' = N$).

Let $D = (d_1, \dots, d_N)$ a population-scale dataset of size $N > 1$, with records d_i in some arbitrary set $\mathcal{D}$. A *matching* relation $\mathcal{M} : D \times D \to \{0, 1\}$ is defined over records in D, that outputs 1 iff two records are identical. This relation defines k equivalence classes $\mathcal{C}_1, \dots, \mathcal{C}_k$ of sizes $n_1, \dots, n_k$, such that $i, j \in \mathcal{C}_l \Leftrightarrow \mathcal{M}(d_i, d_j) = 1$. We show below that this list of class sizes $(n_1, \dots, n_k)$ uniquely characterises the behavior of the uniqueness in a sample of size $m < N$.

A dataset $D' \subset D$ of size $m \leq N$ (the sample dataset) is sampled uniformly without replacement from D. For each record d_i in D, we define the binary random variable $S_i = I\{d_i \in D'\}$. S_i follows a Bernoulli distribution with parameter $\frac{m}{N}$, and all Y_i satisfy the following equation: $\sum_{i=1}^{N} Y_i = m$. From this, we define the *count per class* variable $N_j = \sum_{i \in C_j} S_i$ for $j \in \{1, \dots, k\}$.

Using these notations, the sample uniqueness for a sample size $m \leq N$ is defined as the expected fraction of records of D' that are unique in D', i.e. that are the only records of their class in D':

$$\nu_D(m) = \mathbb{E}\left[\frac{1}{m}\sum_{i=1}^{k} I\{N_i = 1\}\right] = \frac{1}{m}\sum_{i=1}^{k} \mathbb{P}\left[N_i = 1\right]$$

Denote by $\binom{a}{b}$ the number of subsets of size a of a set of size b ("a choose b"). The probability that $N_i = 1$ for some class of size n_i is equal to the fraction of samples of size m from the dataset (of size N) that have exactly 1 record from the class:

$$\mathbb{P}\left[N_i = 1\right] = \frac{\binom{1}{n_i} \cdot \binom{m-1}{N-n_i}}{\binom{m}{N}} = \frac{n_i \cdot \frac{(N-n_i)!}{(m-1)!\ (N-n_i-m+1)!}}{\frac{N!}{m!\ (N-m)!}} = n_i \cdot m \cdot \frac{(N-n_i)!}{N!} \cdot \prod_{j=0}^{n_i-2}(N-m-j)$$

We then use this expression to compute the sample uniqueness:

$$\nu_D(m) = \frac{1}{m}\sum_{i=1}^{k} m\, n_i \frac{(N-n_i)!}{N!} \prod_{j=0}^{n_i-2}(N-m-j) = \sum_{i=1}^{k} n_i \frac{(N-n_i)!}{N!} \cdot \prod_{j=0}^{n_i-2}(N-m-j)$$

We show that ν_D is a convex function of m, for $m \in [1, N]$, for any dataset D of size N. For this, observe that $\nu_D(m)$ is a linear combination of the convex function $\hat{f}_{n_i}(m)$ as defined in Lemma 7.1, for $i = 1, \dots, k$, with nonnegative linear coefficients. We show in Lemma 7.1 that $\hat{f}_{n_i}$ is a convex

function of m for all n_i, and thus, $\nu_D(m)$ is a convex, decreasing, function of m.

Lemma 7.1 Let $n_i \in \mathbb{N}_0$, $N > n_i$. The function $\hat{f}_{n_i}$ defined as:

$$\hat{f}_{n_i} : [0, N] \to \mathbb{R} : m \mapsto \begin{cases} \prod_{j=0}^{n_i-2}(N - m - j) & \text{if } m \leq N - n_i + 2 \\ 0 & \text{if } m > N - n_i + 2 \end{cases}$$

is a convex, decreasing, non-negative function of m.

Proof. First, Define the function $f_{n_i} : \mathbb{R}^+ \to \mathbb{R} : m \mapsto \prod_{j=0}^{n_i-2}(N - m - j)$. We show that $\forall\, m$ such that $N \geq m + n_i - 2$, the first derivative is non-positive, and the second derivative of f_{n_i} is non-negative:

$$\begin{cases} \frac{\mathrm{d}f_{ni}}{\mathrm{d}m}(m) = (-1) \cdot \sum_{l=0}^{n_i-2} \prod_{j \neq l}(N - m - j) \leq 0 \\ \frac{\mathrm{d}^2 f_{ni}}{\mathrm{d}m^2}(m) = \sum_{l=0}^{n_i-2} \sum_{j \neq l} \prod_{s \neq j, s \neq l}(N - m - s) \geq 0 \end{cases}$$

and thus f_{n_i} is decreasing and convex over $[0, m + n_i - 2]$. From this, one gets that $\hat{f}_{n_i}$ is also a decreasing non-negative function over $[0, N]$. Further, since $f(N - n_i + 2) = 0$, this function is continuous.

We now show that $\hat{f}_{n_i}$ satisfies the definition of convexity over $[0, N]$: $\forall x, y \in \mathbb{R}^+$ with $x \leq y$, and $\forall \lambda \in [0, 1]$, $\hat{f}_{n_i}(\lambda x + (1 - \lambda)y) \leq \lambda x + (1 - \lambda)y$. Denote $z = \lambda x + (1 - \lambda)y$, and $\kappa = N - n_i + 2$, and observe that:

- Either $x \leq y \leq \kappa$, and the inequality holds by convexity of f_{n_i} over $[0, \kappa]$;
- Either $\kappa \leq x \leq y$, and the inequality holds since $\hat{f}_{n_i}(x) = \hat{f}_{n_i}(z) = \hat{f}_{n_i}(y) = 0$.
- Either $x < \kappa \leq z \leq y$, and the inequality holds since $\hat{f}_{n_i}(x) > 0$ and $\hat{f}_{n_i}(y) = \hat{f}_{n_i}(z) > 0$.
- Either $x \leq z < \kappa \leq y$. Then, there exists $\mu \in [0, 1]$ such that $z = \mu x + (1 - \mu)\kappa$. By convexity of f_{n_i}, we have $\hat{f}_{n_i}(z) \leq \mu \hat{f}_{n_i}(x) + (1 - \mu) f_{n_i}(\kappa) = \mu \hat{f}_{n_i}(x)$. Further, observe that $z = \mu x + (1 - \mu)\kappa = \lambda x + (1 - \lambda)y \geq \lambda x + (1 - \lambda)\kappa$, and thus $\lambda \geq \mu$. Combined together, this implies $\hat{f}_{n_i}(z) \leq \mu \hat{f}_{n_i}(x) \leq \lambda \hat{f}_{n_i}(x) = \lambda \hat{f}_{n_i}(x) + (1 - \lambda)\hat{f}_{n_i}(y)$, since $\hat{f}_{n_i}(x) > 0$ and $\hat{f}_{n_i}(y) = 0$.

□

We then compute the sample uniqueness of the dataset used by El Emam's method, which we call D_α. By construction, this dataset has $\alpha N \in \mathbb{N}$ classes of size $n_i = 1,\ i = 1, \ldots, \alpha N$, and $\frac{(1-\alpha)N}{2}$ classes of size $n_i = 2,\ i = \alpha N + 1, \ldots, k$. We thus get:

$$\nu_{D_\alpha}(m) = \alpha N \cdot \frac{1}{N} + \frac{(1 - \alpha)N}{2} \cdot 2\frac{N - m}{N(N - 1)} = \alpha + (1 - \alpha)\left(1 - \frac{m - 1}{N - 1}\right)$$

which is an affine function of m.

Let (m^*, ε^*) an empirical measurement of uniqueness on a dataset of size m^*, assumed to be a uniform sample from a sample of size N. We show that if there exists an α for which $\nu_{D_\alpha}(m^*) = \varepsilon^*$, then *any* other dataset D of size N such that $\nu_D(m^*) = \varepsilon^*$ will have higher sample uniqueness for any $m' \geq m^*$: $\nu_D(m') \geq \nu_{D_\alpha}(m')$. This is a direct consequence of the convexity of $\nu_D(m)$ and linearity of $\nu_{D_\alpha}(m)$. Indeed, convexity for $\nu_D(m)$ implies that, $\forall m' \geq m^*$ (using the definition with $x = m'$, $y = 1$ and $\lambda' = \frac{m^*-1}{m'-1} \leq 1$):

$$\varepsilon^* = \nu_D(m^*) \leq \lambda' \cdot \nu_D(m') + (1 - \lambda') \cdot \nu_D(1)$$

which in turn entails, since $\nu_D(1) = 1$:

$$\nu_D(m') \geq \frac{\varepsilon^* - 1 + \lambda'}{\lambda'} = \frac{m' - 1}{m^* - 1}(\varepsilon^* - 1) + 1$$

Observe that choosing $\alpha^* = 1 + \frac{N-1}{m^*-1}(\varepsilon^* - 1)$ in El Emam's method, if $\alpha \leq 1$, is such that $\nu_{D_{\alpha^*}}(m^*) = \varepsilon^*$. If $\alpha > 1$, then El Emam's method cannot be used. We then get, for all $m' \geq m^*$:

$$\nu_{D_{\alpha^*}}(m') = \frac{m' - 1}{m^* - 1}(\varepsilon^* - 1) + 1$$

And thus, for *any* dataset D of size N, if α exists, we have that, $\forall\, m' \geq m^*$:

$$\nu_{D_{\alpha^*}}(m') \leq \nu_D(m')$$

8 A naive unicity model: the combinatorial approach

A naive approach to model unicity is to approach it as a purely combinatorial problem with every time and location pair being equally likely to appear in a trace.

Given N trajectories of length λ hours, the model assigns to each of the λ hours a particular location $\mathcal{L}_i$ from the $|\mathcal{L}|$ possible locations uniformly at random.

Given p randomly picked points $A_p^{D_i}$ from a trajectory D_i, the probability of D_i being uniquely identifiable given $A_p^{D_i}$ is equal to the probability of all other $N-1$ trajectories not containing all of $A_p^{D_i}$:

$$\mathbb{P}\left[A_p^{D_i} \not\subseteq D_j \;\forall\, j \neq i\right] = \prod_{i \neq j}^{N} \left(1 - \mathbb{P}[A_p^{D_i} \subseteq D_j]\right) \tag{S32}$$

The probability $P[A_p^{D_i} \subseteq D_j]$ can be computed using the fact that the probability of each of the $|\mathcal{L}|$ locations appearing in $A_p^{(i)}$ is equal:

$$\mathbb{P}\left[A_p^{D_i} \not\subseteq D_j\right] = \prod_k^p \mathbb{P}[\mathcal{L}_k \in A_p^{D_i}] = \left(\frac{1}{|\mathcal{L}|}\right)^p \tag{S33}$$

The definition of our unicity estimate is the average of equation S32 over all trajectories. Since the trajectories are independently generated, this equation indeed measures the unicity of the combinatorial model. Using the expression from equation S33 we then have:

$$\begin{aligned}\varepsilon_p^{comb}(N) &= \prod_{i\neq j}^N \left(1 - \mathbb{P}[A_p^{D_i} \subseteq D_j]\right) \\ &= \prod_{i\neq j}^N \left(1 - \frac{1}{|\mathcal{L}|^p}\right) \\ &= \left(1 - \frac{1}{|\mathcal{L}|^p}\right)^{N-1} \end{aligned} \tag{S34}$$

where equation S34 gives a simple form for the naive model.

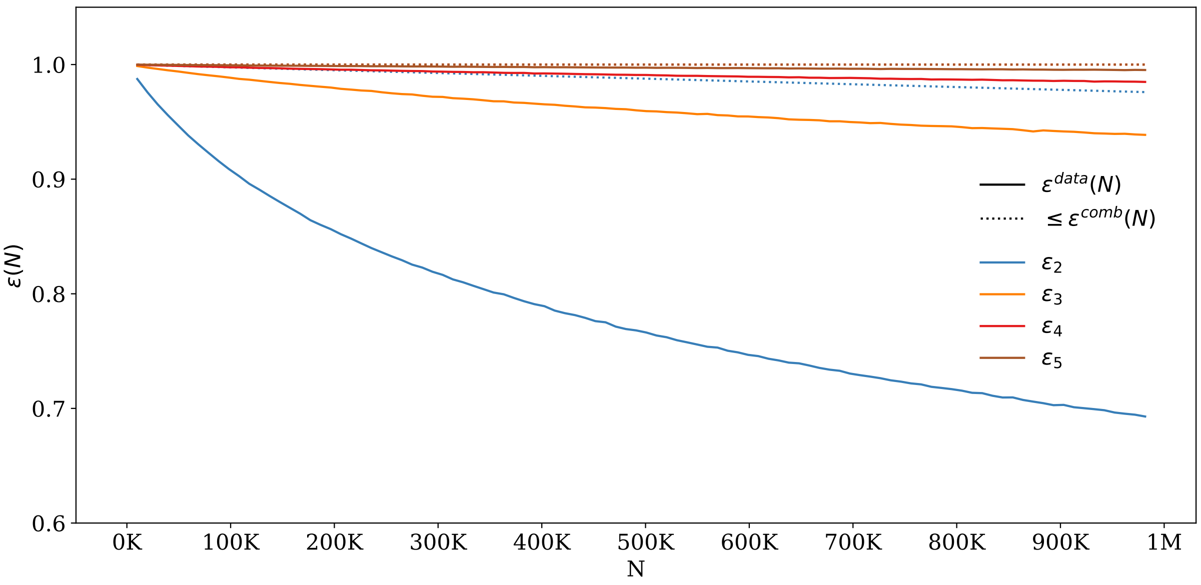

Fig S3 The combinatorial unicity model (dotted lines) greatly overestimates empirical unicity (solid lines).

The combinatorial unicity model estimates are compared to empirical unicity in Figure S3. It is clear that the combinatorial model greatly overestimates unicity values with the dotted lines hugging the region of $\varepsilon \approx 1$ for nearly all values of p.

9 Dependence of the unicity model on input distributions

For an expanded discussion see the main text.

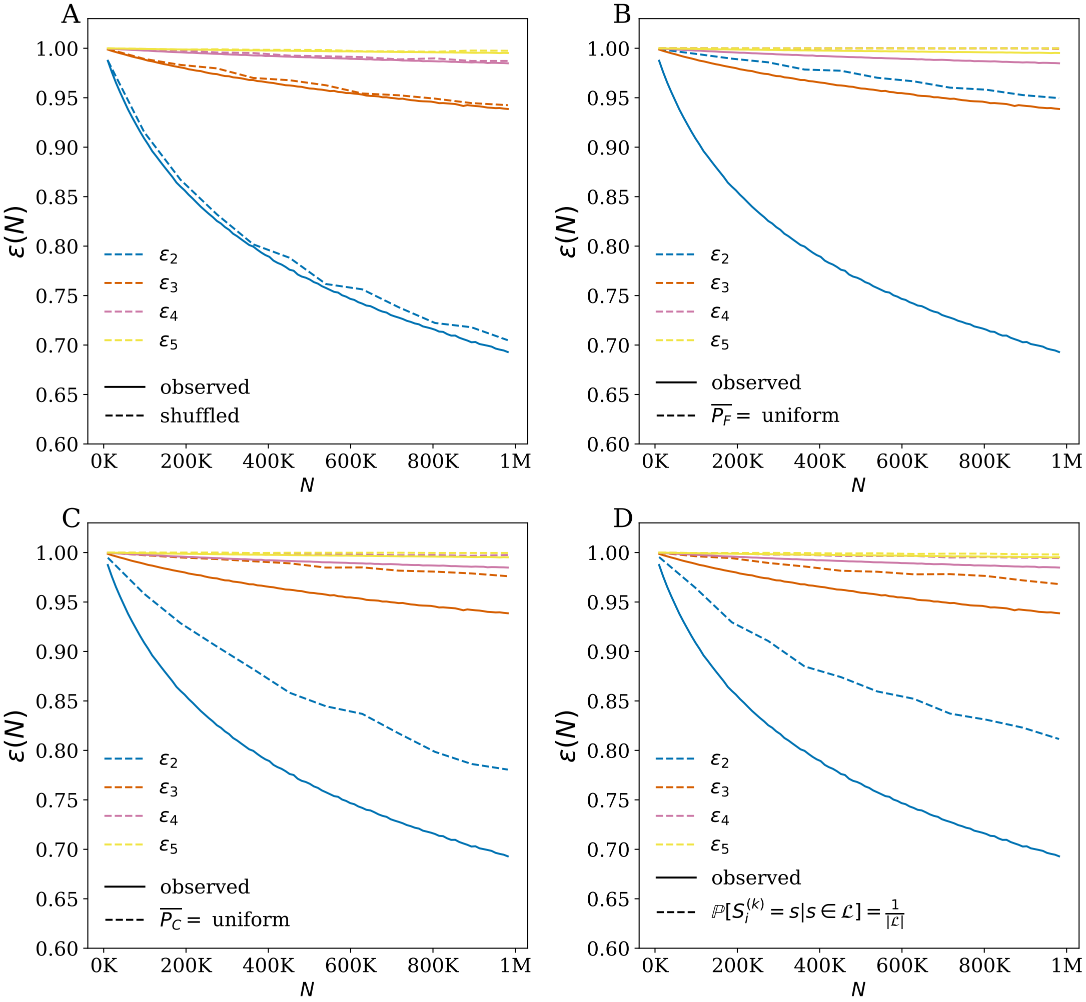

Fig S4 Unicity of alternative models compared to observed unicity. **A:** The space and time components of trajectories are shuffled. The P_F distribution (**B**) and the P_C distribution (**C**) are separately replaced with the uniform distribution. **D:** The antenna set S_i is populated with uniformly picked antennas from $\mathcal{L}$ at random, instead of using the sub-graph sampling method.

10 Convergence of the unicity model

The unicity model extracts a set of 3 distributions (collectively called R_n when computed from data of size (n)) to model the decrease of unicity. We now show how estimates of unicity converge as a function of the size of the data set n from which the distributions are extracted with the following approach:

1. We instantiate the unicity model using input distributions R_n with increasing n as sampled uniformly from the data set of 1M trajectories
2. We use each instantiation to obtain unicity values for data sets ranging from 10K to 1M trajectories. This gives a vector of unicity values
3. Finally, we compare each of these unicity vectors, to the vector obtained by a model instantiated using the entire data (i.e. $n = 1M$) using the Root Mean Squared Error of the vectors.

Figure S5 shows that for varying values of n (the horizontal axis), the instantiations of the unicity model quickly converge to the model with $n = 1M$. Indeed, the rate at which the input distributions converge is such that even with only $n = 2.5K$ ($\approx$ $\%0.0025$ of the data) the results of the unicity models are largely the same as a unicity model that used all 1M trajectories as its input (stable RMSE values). Finally, lines of best fit are also included showing almost horizontal lines across the board.

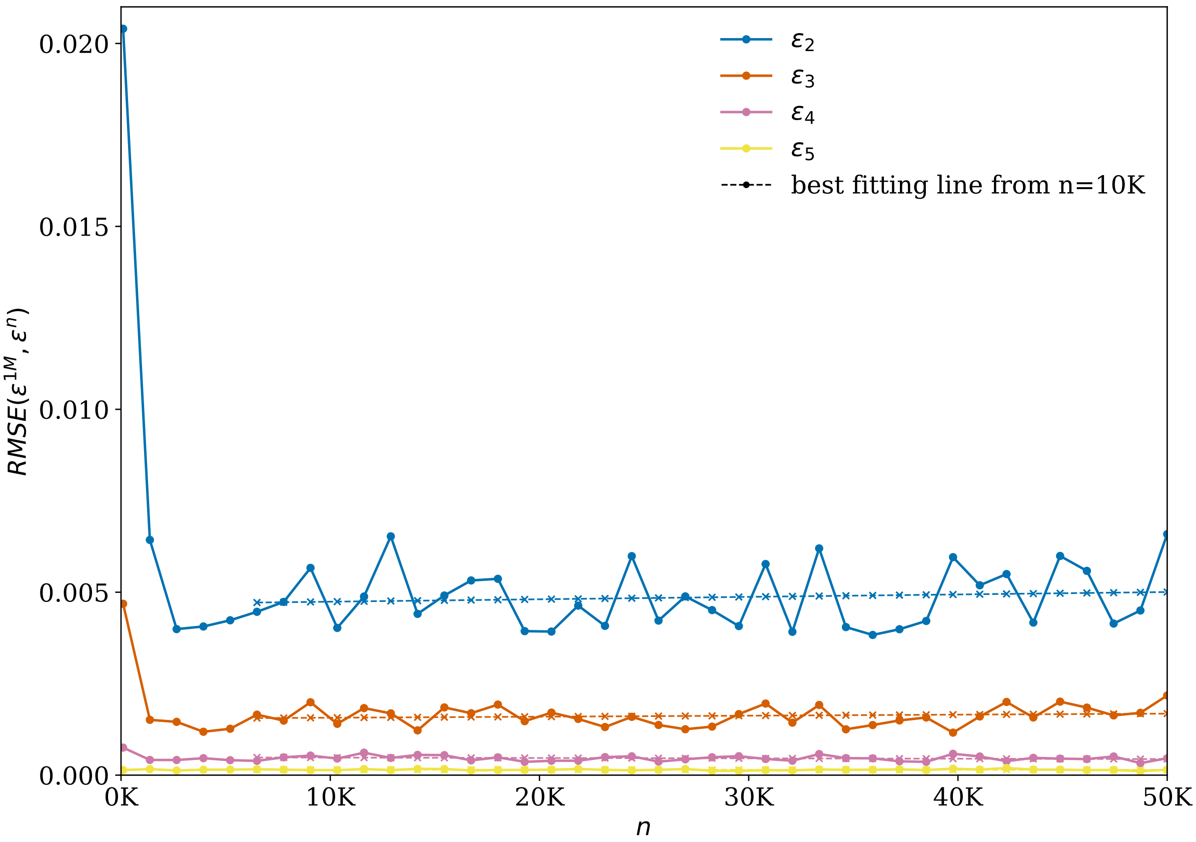

Fig S5 Convergence of the unicity model as the size of the sample (n) from which the input distributions are extracted increases. In solid, the RMSE (vertical axis) of unicity models instantiated with distributions extracted from varying number of trajectories (horizontal axis) is displayed. The dotted line is the best fitting line to each learning curve starting from n=10,000.
